# Metal-Free Regiodivergent Addition of Carbon Nucleophiles to α,β-Unsaturated Electrophiles

**DOI:** 10.3390/molecules22071178

**Published:** 2017-07-14

**Authors:** Cédric Spitz, Alain G. Giuglio-Tonolo, Thierry Terme, Patrice Vanelle

**Affiliations:** Aix-Marseille University, CNRS, ICR UMR 7273, Equipe Pharmaco-Chimie Radicalaire, Faculté de Pharmacie, 27 Boulevard Jean Moulin, CS 30064, 13385 Marseille CEDEX 05, France; gamal.giuglio-tonolo@univ-amu.fr (A.G.G.-T.); thierry.terme@univ-amu.fr (T.T.)

**Keywords:** metal-free, regiodivergent, α,β-unsaturated electrophiles

## Abstract

A mild and metal-free regiodivergent addition of carbon nucleophiles to α,β-unsaturated electrophiles was developed. Total 1,2-regioselectivity was observed in the addition of nitrobenzyl chloride derivative **1** to α,β-unsaturated aldehydes **2** in the presence of TDAE. Moreover, the reaction between *p*-nitrobenzyl chloride **1a** and α,β-unsaturated iminium salts **4** led to the formation of the 1,4-adduct with total regioselectivity.

## 1. Introduction

The conjugate addition of carbanions to α,β-unsaturated carbonyl compounds is one of the most fundamental carbon–carbon bond-forming reactions in organic synthesis [1,2,3,4,5,6]. When a carbon nucleophile is added to α,β-unsaturated aldehydes, it can lead to 1,2-addition or 1,4-addition. This depends on several factors, such as the nature of both organometallic species and substrates. As a general rule, organolithiums are considered the best reagents to promote 1,2-addition, whereas organocuprates predominantly promote conjugate addition.

Tetrakis(dimethylamino)ethylene (TDAE) is an organic reducing agent that reacts with halogenated derivatives to generate a carbanion under mild conditions via a charge transfer complex [7,8,9]. In particular, we have shown that from *o*- and *p*-nitrobenzyl chlorides, tetrakis (dimethylamino)ethylene (TDAE) is able to generate a nitrobenzyl carbanion that can react with various electrophiles such as aromatic aldehydes, α-ketoester, ketomalonate, α-ketolactam, and imine derivatives [10,11,12,13,14,15,16,17,18]. More recently, we showed that using TDAE for the regioselective addition of *p*-nitrobenzyl chloride or 2,3-bis(bromomethyl)quinoxaline to α,β-unsaturated tosylimines allowed the synthesis of allylamines in good yields and with total 1,2-regioselectivity [19]. Here, our aim was to determine how the carbanion generated by our metal-free conditions would react in the presence of α,β-unsaturated aldehydes.

## 2. Results and Discussion

First, the reaction between *p*-nitrobenzyl chloride **1a** and *trans*-cinnamaldehyde **2a** in the presence of TDAE was selected as a model (Table 1). Using 1 equivalent of *p*-nitrobenzyl chloride **1a**, 1 equivalent of TDAE and 1.2 equivalent of *trans*-cinnamaldehyde **2a** in acetonitrile gave a low 33% yield of compound **3aa** (Entry 1). Interestingly, however, total 1,2-regioselectivity was observed. Using two equivalents of aldehyde allowed the synthesis of **3aa** in 48% yield (Entry 2). Using DMF instead of acetonitrile or changing the order of addition slightly decreased the reactivity (Entry 3 and 4). A better 53% yield was obtained using two equivalents of *p*-nitrobenzyl chloride **1a**, two equivalents of TDAE, and one equivalent of *trans*-cinnamaldehyde **2a** (Entry 5).

Interestingly, there is no need to decrease temperature to −20 °C, as the yield was slightly better at room temperature (Entry 6). More equivalents of **1a** and TDAE were beneficial to the reaction (Entry 7). A sequential addition of both *p*-nitrobenzyl chloride **1a** and TDAE afforded alcohol **3aa** in a good 67% yield (Entry 8). So, the best reaction conditions were three equivalents of *p*-nitrobenzyl chloride **1a**, three equivalents of TDAE, and one equivalent of *trans*-cinnamaldehyde **2a** in acetonitrile at room temperature, with slow addition of **1a** over 30 min (Entry 9).

**Scheme 1:**
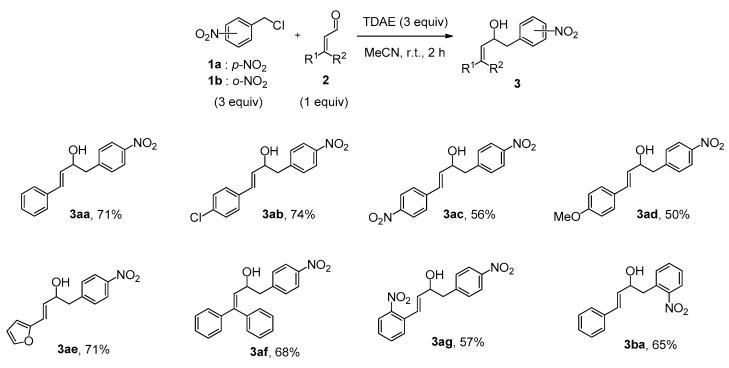
Regioselective 1,2-addition of nitrobenzyl chloride derivative **1** to various cinnamaldehyde **2** in the presence of TDAE.

With the optimized conditions in hand, we next investigated the generality and scope of the reaction with a series of α,β-unsaturated aldehydes **2a**–**g** (Scheme 1). Both electron-poor (**3ab** and **3ac**) and electron-rich substituents (**3ad**) gave good yields and total 1,2-regioselectivities. Interestingly, the reaction conditions are tolerant towards the formation of alcohol **3ae** from heteroaromatic aldehyde **2e**.

Moreover, the more bulky β-disubstituted aldehyde **2f** gave only the product of 1,2-addition **3af** in 68% yield. Ortho substituents on the aldehyde (**3ag**) and on the benzyl chloride derivative (**3ba**) were both well tolerated.

Thus, in all cases, the carbanion generated by our metal-free conditions gave only the 1,2-regioisomer when reacting with α,β-unsaturated aldehydes. Could the reactivity be changed from 1,2 to 1,4-addition? As organocuprates predominantly promote conjugate addition, we tested different conditions using copper salts but no reaction occurred, probably due to problems of compatibility between TDAE and copper derivatives.

**Scheme 2:**
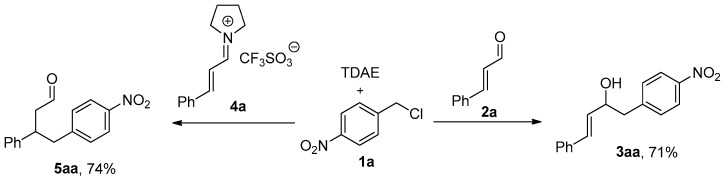
Regiodivergent addition of *p*-nitrobenzyl chloride **1a** to α,β-unsaturated electrophiles.

To our satisfaction, however, using α,β-unsaturated iminium salts instead of aldehydes completely changed the regioselectivity of the addition. Indeed, the reaction between *p*-nitrobenzyl chloride **1a** and (*E*)-1-(3-phenylallylidene)pyrrolidin-1-ium **4a** in the presence of TDAE led to the formation of the 1,4-adduct **5aa** alone, in a very good 74% yield (Scheme 2).

A general feature that emerges from the ^13^C-NMR studies of unsaturated imines is the observed downfield shift (ca. 5–10 ppm) at C^3^ in iminium salts compared to the parent unsaturated aldehydes. This feature might reflect the increased electrophilicity of the iminium cation at C^3^ compared to the parent aldehyde [20].

This metal-free controlled regiodivergence is of great interest, because the same *p*-nitrobenzyl chloride **1a** can lead to the formation of both 1,2 and 1,4-adduct without requiring metallic species like organolithiums for 1,2-addition or organocuprates for 1,4-addition.

**Scheme 3:**
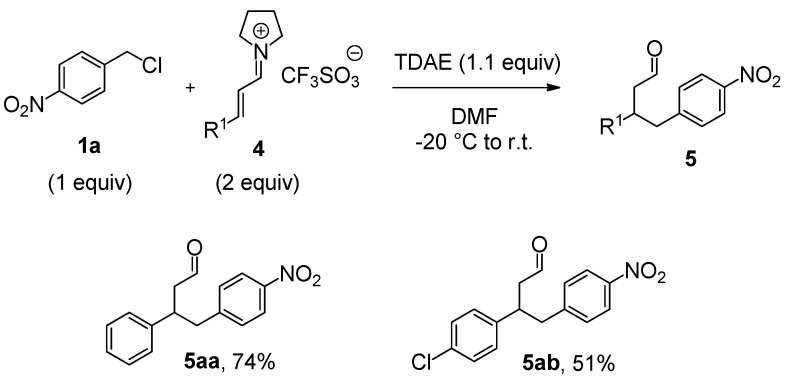
Regioselective 1,4-addition of *p*-nitrobenzyl chloride **1a** to α,β-unsaturated iminium salt **4** in the presence of TDAE.

We next investigated the possibility of using another α,β-unsaturated iminium salt **4b** (Scheme 3). Here, too, total 1,4-regioselectivity was observed and allowed the synthesis of the aldehyde **5ab** in 51% yield.

**Scheme 4:**
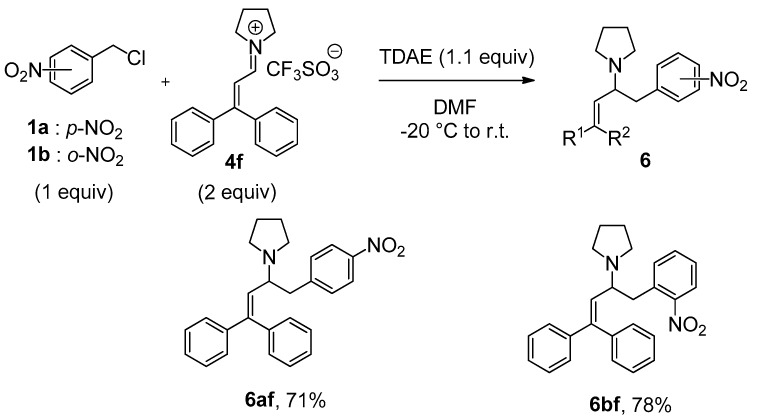
Regioselective 1,2-addition of nitrobenzyl chloride derivative **1** to bulky α,β-unsaturated iminium salt **4f** in the presence of TDAE.

By contrast with the previous examples, the reaction between *p*-nitrobenzyl chloride **1a** and the more bulky β-disubstituted iminium salt **4f** gave only the 1,2-adduct **6af**, probably due to steric hindrance at the β-position (Scheme 4). We next turned our attention to the use of *o*-nitrobenzyl chloride **1b** instead of *p*-nitrobenzyl chloride **1a**. As expected, the reaction between *o*-nitrobenzyl chloride **1b** and the bulky β-disubstituted iminium salt **4f** also gave only the 1,2-adduct **6bf** with a very good 78% yield.

## 3. Experimental Section

### 3.1. General

Melting points were determined on a Büchi melting point B-540 apparatus (BÜCHI Labortechnik AG, Flawil, Switzerland) and are uncorrected. Element analyses were performed on a Thermo Finnigan EA1112 (San Jose, CA, USA) at the spectropole of Aix-Marseille University. Both ^1^H- and ^13^C-NMR spectra were determined on a Bruker AC 250 spectrometer (Wissembourg, France) at the Service de RMN de la Faculté de Pharmacie de Marseille of the Aix-Marseille University. The ^1^H and the ^13^C chemical shifts are reported from CDCl_3_ peaks: ^1^H (7.26 ppm) and ^13^C (77.16 ppm). Multiplicities are represented by the following notations: s, singlet; d, doublet; t, triplet; q, quartet; m, a more complex multiplet or overlapping multiplets. The following adsorbents were used for column chromatography: Silica gel 60 (Merck, Darmstadt, Germany, particle size 0.063–0.200 mm, 70–230 mesh ASTM). TLC was performed on 5 × 10 cm aluminum plates coated with silica gel 60 F_254_ (Merck, Darmstadt, Germany) in an appropriate solvent.

### 3.2. General Procedure for the Synthesis of Alcohols ***3***

Under nitrogen atmosphere at room temperature, to a stirred solution of α,β-unsaturated aldehydes **2** (0.2 mmol) in MeCN (1 mL) was added TDAE (140 µL, 0.6 mmol) followed by addition of a solution of the nitrobenzyl chloride derivative **1** (103 mg, 0.6 mmol) in MeCN (1 mL) over 30 min. Reactions were stirred at RT for 2 h. Water (10 mL) was added and the aqueous solution was extracted with CH_2_Cl_2_ (3 × 10 mL). The combined organic layer was washed with brine (20 mL) and dried over MgSO_4_. Evaporation of the solvent furnished the crude product. Purification by silica gel chromatography (PE/EtOAc: From 9/1 to 6/4 depending on the polarity of substrates) afforded pure alcohol products **3**.

The general procedure was followed with **2a** (26 mg) and **1a** (130 mg). *(E)-1-(4-nitrophenyl)-4-phenylbut-3-en-2-ol* (**3aa**). 71% yield; yellow solid; m.p. 94–96 °C; ^1^H-NMR (250 MHz, CDCl_3_) δ 8.16 (d, *J* = 8.6 Hz, 2H), 7.42 (d, *J* = 8.6 Hz, 2H), 7.35–7.26 (m, 5H), 6.58 (d, *J* = 15.9 Hz, 1H), 6.24 (dd, *J* = 15.9, 6.6 Hz, 1H), 4.61–4.53 (m, 1H), 3.03 (d, *J* = 5.5 Hz, 2H), 1.93 (s, 1H). ^13^C-NMR (63 MHz, CDCl_3_) δ 146.8, 145.9, 136.2, 131.4, 130.8, 130.6, 128.8, 128.1, 126.6, 123.7, 73.3, 43.7; HRMS (ESI): *m*/*z* [M + Na]^+^ calcd. for [C_16_H_15_NO_3_Na]^+^: 292.0944; found: 292.0945.

The general procedure was followed with **2b** (33 mg) and **1a** (130 mg). *(E)-4-(4-chlorophenyl)-1-(4-nitrophenyl)but-3-en-2-ol* (**3ab**). 74% yield; yellow solid; m.p. 112–114 °C; ^1^H-NMR (250 MHz, CDCl_3_) δ 8.17 (d, *J* = 8.7 Hz, 2H), 7.42 (d, *J* = 8.6 Hz, 2H), 7.28–7.24 (m, 4H), 6.53 (d, *J* = 15.9, 1H), 6.22 (dd, *J* = 15.9, 6.5 Hz, 1H), 4.60–4.53 (m, 1H), 3.04–3.01 (m, 2H), 1.78 (s, 1H). ^13^C-NMR (63 MHz, CDCl_3_) δ 146.9, 145.8, 134.8, 133.8, 131.4, 130.6, 130.1, 129.0, 127.8, 123.7, 73.2, 43.7; HRMS (ESI): *m*/*z* [M + Na]^+^ calcd. for [C_16_H_14_NO_3_ClNa]^+^: 326.0554; found: 326.0555.

The general procedure was followed with **2c** (35 mg) and **1a** (130 mg). *(E)-1,4-bis(4-nitrophenyl)but-3-en-2-ol* (**3ac**). 56% yield; yellow solid; m.p. 111–113 °C; ^1^H-NMR (250 MHz, DMSO) δ 8.19–8.13 (m, 4H), 7.68 (d, *J* = 8.9 Hz, 2H), 7.54 (d, *J* = 8.7 Hz, 2H), 6.66 (s, 2H), 5.36 (s, 1H), 4.49 (s, 1H), 3.03 (dd, *J* = 13.4, 5.1 Hz, 1H), 2.90 (dd, *J* = 13.4, 7.7 Hz, 1H). ^13^C-NMR (63 MHz, DMSO) δ 147.3, 146.2, 146.0, 143.6, 138.7, 130.9, 127.2, 126.8, 124.0, 123.1, 71.1, 42.9; HRMS (ESI): *m*/*z* [M + Na]^+^ calcd. for [C_16_H_14_N_2_O_5_Na]^+^: 337.0795; found: 337.0797.

The general procedure was followed with **2d** (32 mg) and **1a** (130 mg). *(E)-4-(4-methoxyphenyl)-1-(4-nitrophenyl)but-3-en-2-ol* (**3ad**). 50% yield; yellow solid; m.p. 115–117 °C; ^1^H-NMR (250 MHz, CDCl_3_) δ 8.17 (d, *J* = 8.6 Hz, 2H), 7.42 (d, *J* = 8.6 Hz, 2H), 7.29 (d, *J* = 8.7 Hz, 2H), 6.86 (d, *J* = 8.7 Hz, 2H), 6.51 (d, *J* = 15.9 Hz, 1H), 6.09 (dd, *J* = 15.9, 6.9 Hz, 1H), 4.58–4.51 (m, 1H), 3.81 (s, 3H), 3.03 (d, *J* = 5.7 Hz, 2H), 1.68 (s, 1H). ^13^C-NMR (63 MHz, CDCl_3_) δ 159.7, 146.9, 146.1, 131.1, 130.6, 128.9, 128.5, 127.9, 123.7, 114.2, 73.6, 55.5, 43.8; HRMS (ESI): *m*/*z* [M + Na]^+^ calcd. for [C_17_H_17_NO_4_Na]^+^: 322.1050; found: 322.1050.

The general procedure was followed with **2e** (24 mg) and **1a** (130 mg). *(E)-4-(furan-2-yl)-1-(4-nitrophenyl)but-3-en-2-ol* (**3ae**). 71% yield; yellow solid; m.p. 67–69 °C; ^1^H-NMR (250 MHz, CDCl_3_) δ 8.17 (d, *J* = 8.2 Hz, 2H), 7.44–7.36 (m, 3H), 6.44–6.38 (m, 2H), 6.25–6.14 (m, 2H), 4.55–4.50 (m, 1H), 3.08–2.99 (m, 2H), 1.72 (s, 1H). ^13^C-NMR (63 MHz, CDCl_3_) δ 151.9, 146.9, 145.8, 142.5, 130.6, 129.2, 123.7, 119.6, 111.5, 108.9, 72.9, 43.7; HRMS (ESI): *m*/*z* [M + Na]^+^ calcd. for [C_14_H_13_NO_4_Na]^+^: 282.0737; found: 282.0738.

The general procedure was followed with **2f** (42 mg) and **1a** (130 mg). *1-(4-nitrophenyl)-4,4-diphenylbut-3-en-2-ol* (**3af**). 68% yield; yellow solid; m.p. 93–95 °C; ^1^H-NMR (250 MHz, CDCl_3_) δ 8.11 (d, *J* = 8.6 Hz, 2H), 7.35–7.17 (m, 10H), 7.01–6.98 (m, 2H), 6.08 (d, *J* = 9.2 Hz, 1H), 4.50–4.41 (m, 1H), 3.07–2.98 (m, 2H), 1.70 (s, 1H). ^13^C-NMR (63 MHz, CDCl_3_) δ 146.8, 145.9, 144.6, 141.2, 139.0, 130.6, 129.44, 129.41, 128.5, 128.4, 128.1, 127.8, 127.5, 123.6, 70.3, 43.8; HRMS (ESI): *m*/*z* [M + Na]^+^ calcd. for [C_22_H_19_NO_3_Na]^+^: 368.1257; found: 368.1255.

The general procedure was followed with 2g (35 mg) and 1a (130 mg). *(E)-4-(2-nitrophenyl)-1-(4-nitrophenyl)but-3-en-2-ol* (**3ag**). 57% yield; yellow solid; m.p. 118–120 °C; ^1^H-NMR (250 MHz, CDCl_3_) δ 8.17 (d, *J* = 8.7 Hz, 2H), 7.93 (d, *J* = 8.0 Hz, 1H), 7.61–7.50 (m, 2H), 7.46–7.38 (m, 3H), 7.05 (d, *J* = 15.7 Hz, 1H), 6.19 (dd, *J* = 15.7, 6.4 Hz, 1H), 4.68–4.60 (m, 1H), 3.07–3.04 (m, 2H), 1.97 (s, 1H). ^13^C-NMR (63 MHz, CDCl_3_) δ 148.0, 147.0, 145.5, 136.2, 133.4, 132.3, 130.6, 128.9, 128.6, 126.8, 124.8, 123.8, 72.8, 43.4; HRMS (ESI): *m*/*z* [M + Na]^+^ calcd. for [C_16_H_14_N_2_O_5_Na]^+^: 337.0795; found: 337.0794.

The general procedure was followed with **2a** (26 mg) and **1b** (130 mg). *(E)-1-(2-nitrophenyl)-4-phenylbut-3-en-2-ol* (**3ba**). 65% yield; yellow solid; m.p. 69–70 °C; ^1^H-NMR (250 MHz, CDCl_3_) δ 7.83 (d, *J* = 8.0 Hz, 1H), 7.48–7.16 (m, 8H), 6.50 (d, *J* = 15.8 Hz, 1H), 6.20 (dd, *J* = 15.9, 6.4 Hz, 1H), 4.56–4.49 (m, 1H), 3.23 (dd, *J* = 13.6, 4.4 Hz, 1H), 3.06 (dd, *J* = 13.6, 8.2 Hz, 1H), 1.94 (s, 1H). ^13^C-NMR (63 MHz, CDCl_3_) δ 150.0, 136.5, 133.6, 133.2, 132.9, 131.2, 130.9, 128.7, 128.0, 127.8, 126.7, 124.9, 73.0, 40.8; HRMS (ESI): *m*/*z* [M + Na]^+^ calcd. for [C_16_H_15_NO_3_Na]^+^: 292.0944; found: 292.0945.

### 3.3. General Procedure for the Synthesis of Iminium Salts ***4***

Compound **4a** was synthesized according to a previously described procedure and experimental data are in accordance with the literature [21].

An aqueous solution of CF_3_SO_3_H (4.00 g in 20 mL of water, 27.0 mmol) was added to a solution of α,β-unsaturated aldehyde **2** (27.0 mmol) and pyrrolidine (2.25 mL, 27.0 mmol) in diethyl ether (25 mL). After 1 h, the organic phase was separated. The aqueous phase was extracted with CH_2_Cl_2_ (15 mL). The organic layers were combined, washed with brine, and dried (MgSO_4_). After evaporation of the solvent under vacuum, the residue was mixed with Et_2_O. The solvent was removed under vacuum, and then the residue was remixed with Et_2_O. This cycle was repeated several times until iminium salt **4** was obtained as a solid.

The general procedure was followed with **2b** (4.5 g). *(E)-1-(3-(4-chlorophenyl)allylidene)pyrrolidin-1-ium trifluoromethanesulfonate (***4b***).* 38% yield; white solid; m.p. 106–108 °C; ^1^H-NMR (250 MHz, CDCl_3_) δ 8.82 (d, *J* = 10.5 Hz, 1H), 7.86 (d, *J* = 15.3 Hz, 1H), 7.63 (d, *J* = 8.6 Hz, 2H), 7.35 (d, *J* = 8.6 Hz, 2H), 7.08 (dd, *J* = 15.3, 10.5 Hz, 1H), 4.15–4.01 (m, 4H), 2.21–2.06 (m, 4H). ^13^C-NMR (63 MHz, CDCl_3_) δ 166.6, 159.4, 139.9, 132.0, 131.7, 129.8, 120.8 (*J*_C,F_ = 321 Hz), 117.9, 57.4, 52.1, 24.7, 24.5; HRMS (ESI): *m*/*z* [M]^+^ calcd. for [C_13_H_15_ClN]^+^: 220.0888; found: 220.0892.

The general procedure was followed with **2f** (5.6 g). *1-(3,3-diphenylallylidene)pyrrolidin-1-ium trifluoromethanesulfonate (***4f***)*. 65% yield; light-blue solid; m.p. 136–138 °C; ^1^H-NMR (250 MHz, CDCl_3_) δ 8.01 (d, *J* = 10.8 Hz, 1H), 7.58–7.35 (m, 10H), 6.90 (d, *J* = 10.8 Hz, 1H), 4.18–4.07 (m, 4H), 2.28–2.13 (m, 4H). ^13^C-NMR (63 MHz, CDCl_3_) δ 171.6, 162.5, 139.1, 136.0, 132.8, 131.64, 131.60, 130.6, 129.12, 129.10, 116.4, 57.9, 52.3, 24.7, 24.5 (CF_3_ not visible under these conditions); HRMS (ESI): *m*/*z* [M]^+^ calcd. for [C_19_H_20_N]^+^: 262.1590; found: 262.1588.

### 3.4. General Procedure for the Synthesis of Aldehydes ***5*** and Allylic Amines ***6***

Under nitrogen atmosphere, TDAE (102 µL, 0.44 mmol) was slowly added, with a syringe, at −20 °C to a vigorously stirred solution of iminium salt **4** (0.80 mmol) with the appropriate nitrobenzyl chloride derivative **1** (69 mg, 0.40 mmol) in 3 mL of DMF. The mixture was then stirred at −20 °C for 45 min and warmed to room temperature for 1 h. Then 10 mL of water was added to quench the reaction. The solution was extracted with dichloromethane (3 × 30 mL), the combined organic layers were washed with brine (3 × 40 mL), and dried over MgSO_4_. The crude product was then obtained after evaporation of the solvent under reduced pressure. Purification by alumina gel chromatography (PE/DCM: From 10/0 to 0/10 depending on the polarity of substrates) afforded the corresponding products **5** or **6**.

The general procedure was followed with **4a** (268 mg) and **1a** (69 mg). *4-(4-nitrophenyl)-3-phenylbutanal (***5aa***)*. 74% yield; yellow oil; ^1^H-NMR (250 MHz, CDCl_3_) δ 9.70 (s, 1H), 8.04 (d, *J* = 8.6 Hz, 2H), 7.29–7.05 (m, 7H), 3.56–3.45 (m, 1H), 3.10 (dd, *J* = 13.3, 5.9 Hz, 1H), 2.95 (dd, *J* = 13.3, 9.2 Hz, 1H), 2.86–2.82 (m, 2H). ^13^C-NMR (63 MHz, CDCl_3_) δ 200.8, 147.3, 146.8, 142.1, 130.1, 128.9, 127.6, 127.3, 123.6, 49.5, 42.9, 41.6; HRMS (ESI): *m*/*z* [M + Na]^+^ calcd. for [C_16_H_15_NO_3_Na]^+^: 292.0944; found: 292.0953.

The general procedure was followed with 4b (296 mg) and 1a (69 mg). *3-(4-chlorophenyl)-4-(4-nitrophenyl)butanal (***5ab***)*. 51% yield; orange oil; ^1^H-NMR (250 MHz, CDCl_3_) δ 9.71 (s, 1H), 8.07 (d, *J* = 8.5 Hz, 2H), 7.24 (d, *J* = 8.5 Hz, 2H), 7.15 (d, *J* = 8.5 Hz, 2H), 7.02 (d, *J* = 8.5 Hz, 2H), 3.56–3.45 (m, 1H), 3.08 (dd, *J* = 16.3, 6.0 Hz, 1H), 2.95–2.84 (m, 3H). ^13^C-NMR (63 MHz, CDCl_3_) δ 200.1, 146.7, 146.6, 140.4, 132.8, 129.9, 128.9, 128.8, 123.6, 49.3, 42.6, 40.8; HRMS (ESI): *m*/*z* [M + Na]^+^ calcd. for [C_16_H_14_NO_3_ClNa]^+^: 326.0560; found: 326.0562.

The general procedure was followed with **4f** (329 mg) and **1a** (69 mg). *1-(1-(4-nitrophenyl)-4,4-diphenylbut-3-en-2-yl)pyrrolidine (***6af***)*. 71% yield; orange oil; ^1^H-NMR (250 MHz, CDCl_3_) δ 8.06 (d, *J* = 8.7 Hz, 2H), 7.25–7.10 (m, 10H), 6.48 (d, *J* = 7.8 Hz, 2H), 6.10 (d, *J* = 10.0 Hz, 1H), 3.31–3.19 (m, 2H), 2.89 (dd, *J* = 13.0, 9.9 Hz, 1H), 2.77–2.69 (m, 4H), 1.86–1.78 (m, 4H). ^13^C-NMR (63 MHz, CDCl_3_) δ 146.8, 146.6, 144.8, 141.5, 139.2, 130.9, 129.4, 128.4, 128.1, 127.8, 127.6, 127.2, 123.4, 64.2, 51.6, 41.3, 23.4 (1 carbon missing due to overlap); HRMS (ESI): *m*/*z* [M + H]^+^ calcd. for [C_26_H_27_N_2_O_2_]^+^: 399.2067; found: 399.2070.

The general procedure was followed with **4f** (329 mg) and **1b** (69 mg). *1-(1-(2-nitrophenyl)-4,4-diphenylbut-3-en-2-yl)pyrrolidine (***6bf***)*. 78% yield; orange oil; ^1^H-NMR (250 MHz, CDCl_3_) δ 7.90 (d, *J* = 8.0 Hz, 1H), 7.48–7.07 (m, 11H), 6.31 (d, *J* = 7.9 Hz, 2H), 6.17 (d, *J* = 10.1 Hz, 1H), 3.70 (dd, *J* = 12.5, 3.5 Hz, 1H), 3.32 (td, *J* = 10.3, 3.5 Hz, 1H), 3.00 (dd, *J* = 12.4, 10.6 Hz, 1H), 2.84–2.74 (m, 4H), 1.87–1.78 (m, 4H). ^13^C-NMR (63 MHz, CDCl_3_) δ 149.7, 144.4, 141.4, 139.1, 134.6, 134.0, 132.8, 129.2, 128.6, 128.3, 128.1, 127.59, 127.57, 127.11, 127.07, 125.0, 63.4, 51.9, 38.2, 23.3; HRMS (ESI): *m*/*z* [M + H]^+^ calcd. for [C_26_H_27_N_2_O_2_]^+^: 399.2067; found: 399.2069.

## 4. Conclusions

In conclusion, regiodivergent addition of nitrobenzyl chloride derivative **1** to α,β-unsaturated electrophiles led to 1,2-addition with α,β-unsaturated aldehydes **2** and 1,4-addition with α,β-unsaturated iminium salts **4**. The mild conditions and the tolerance of nitro groups on the nucleophiles suggest that this method is a good alternative to the use of organometallic reagents to achieve regiodivergent additions on α,β-unsaturated aldehydes. Further research is in progress to extend the scope to a catalytic and asymmetric version using enantiopure organocatalysts.

## Figures and Tables

**Table 1:**
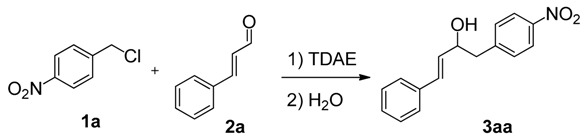
Optimization of the 1,2-addition of *p*-nitrobenzyl chloride **1a** to *trans*-cinnamaldehyde **2a** in the presence of TDAE ^a^.

Entry	1a (Equiv)	2a (Equiv)	TDAE (Equiv)	Solvent	Yield of 3aa (%) ^b^
1	1	1.2	1	MeCN	33
2	1	2	1	MeCN	48
3	1	2	1	DMF	42
4	1	2	1	MeCN	37 ^c^
5	2	1	2	MeCN	53
6	2	1	2	MeCN	55 ^d^
7	3	1	3	MeCN	62 ^d^
8	3	1	3	MeCN	67 ^d,e^
9	3	1	3	MeCN	71 ^d,f^

^a^ Reaction conditions (unless otherwise specified): under nitrogen atmosphere, to a solution of **2a** was added TDAE followed by addition of a solution of **1a**. Reactions were stirred at −20 °C for 1 h and then maintained at room temperature for 2 h; ^b^ Isolated yields; ^c^ To a solution of **2a** and **1a** was added TDAE; ^d^ Reactions were performed at room temperature for 2 h; ^e^ A first 1.5 equiv of TDAE followed by 1.5 equiv of **1a** were added to a solution of **2a**. 1 h later, another 1.5 equiv of TDAE followed by 1.5 equiv of **1a** were added; ^f^ Solution of **1a** was added over 30 min.

## Supplementary Materials

Supplementary Materia are available online, Figures S1–S28: ^1^H- and ^13^C-NMR of all compounds **3aa**–**3ag**, **3ba**, **4b**, **4f**, **5aa**, **5ab**, **6af** and **6bf**.
