# Supplementary material for: Metal-Free Regiodivergent Addition of Carbon Nucleophiles to α,β-Unsaturated Electrophiles
Source: Molecules. 2017 Jul 14;22(7):1178. doi: 10.3390/molecules22071178 (PMC6152382; doi:10.3390/molecules22071178)
Supplement: Supplementary file 1 [file molecules-22-01178-s001.pdf]

Supplementary Materials: **Metal-free regiodivergent addition of carbon nucleophiles to *α*,*β*-unsaturated electrophiles**

Cédric Spitz, Alain G. Giuglio-Tonolo, Thierry Terme and Patrice Vanelle

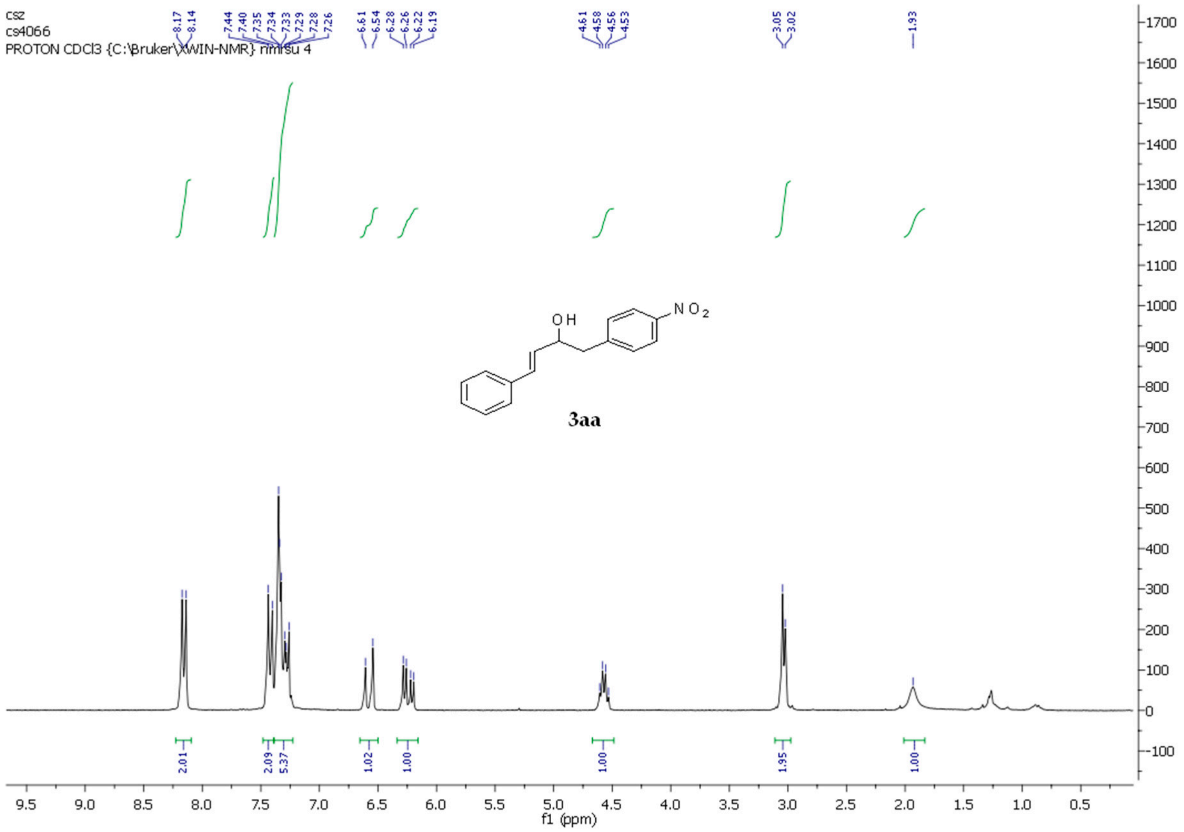

Figure S1. ^{1}H spectra of **3aa**.

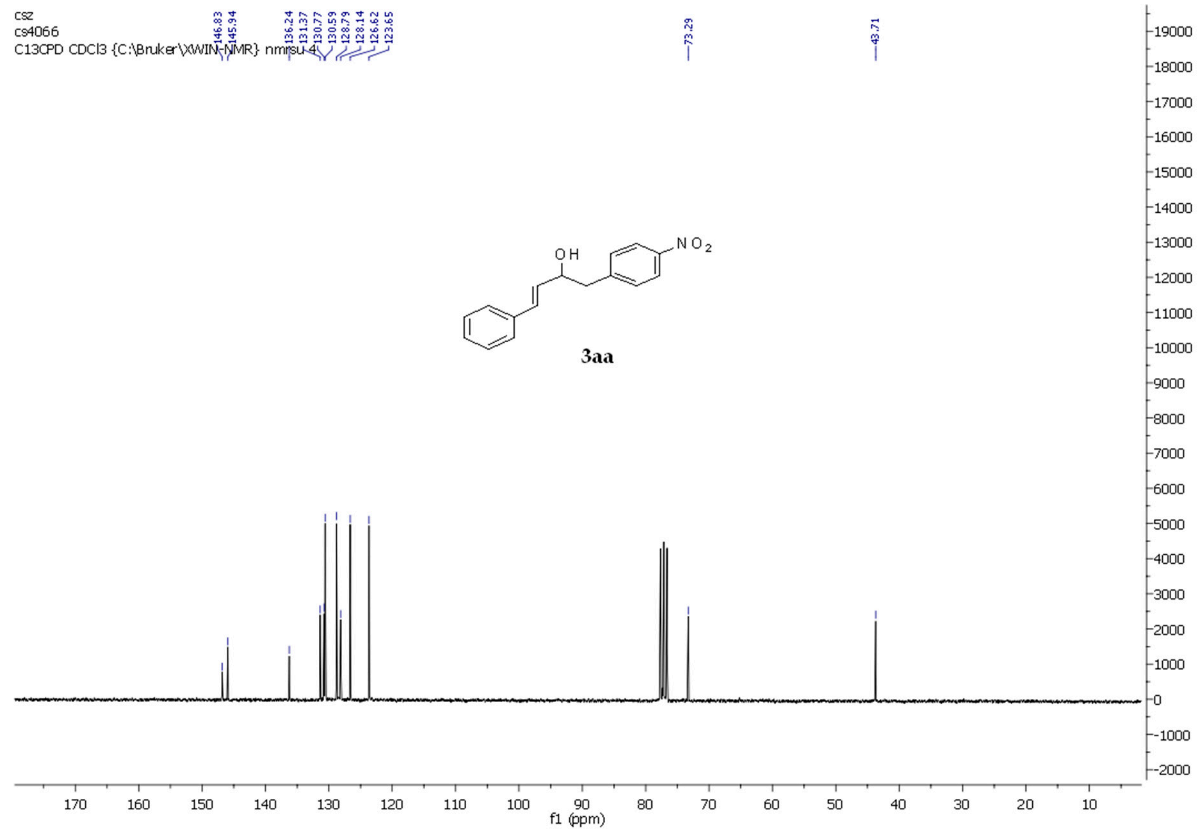

Figure S2. ^{13}C-NMR spectra of **3aa**.

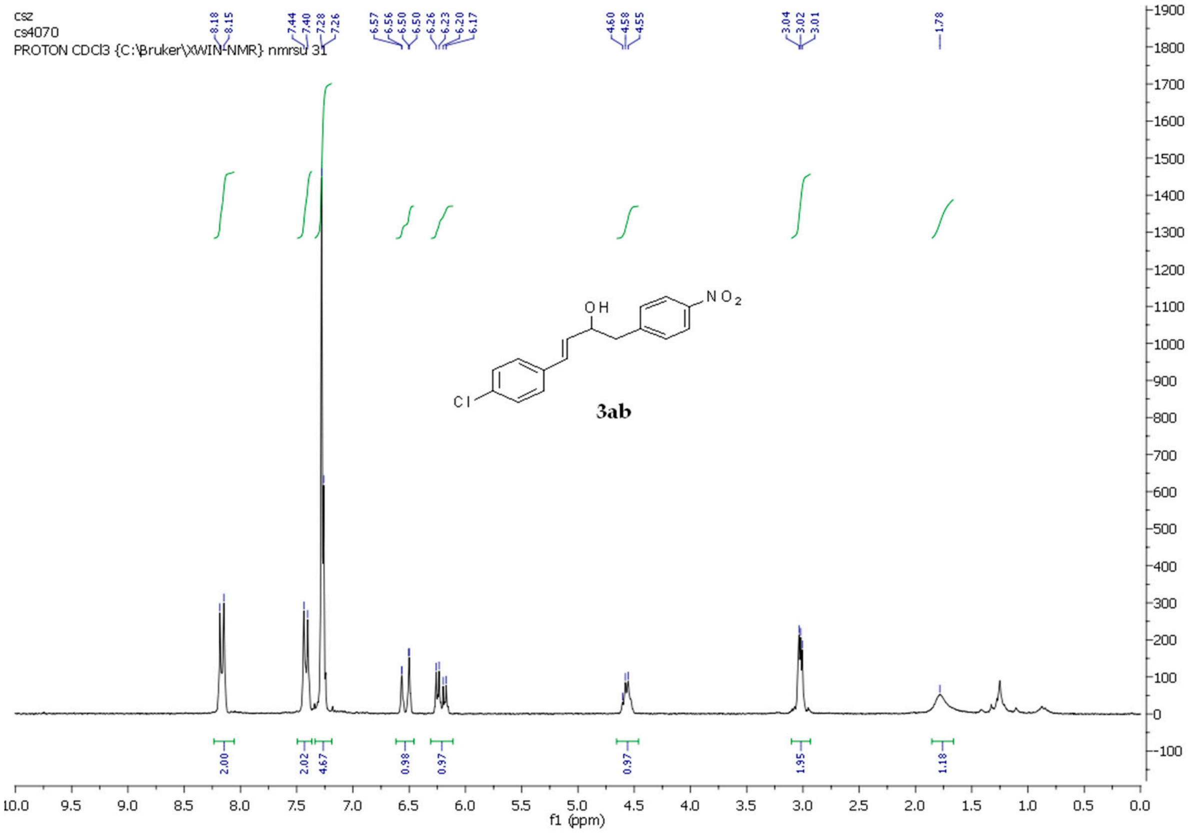

Figure S3. ^{1}H-NMR spectra of **3ab**.

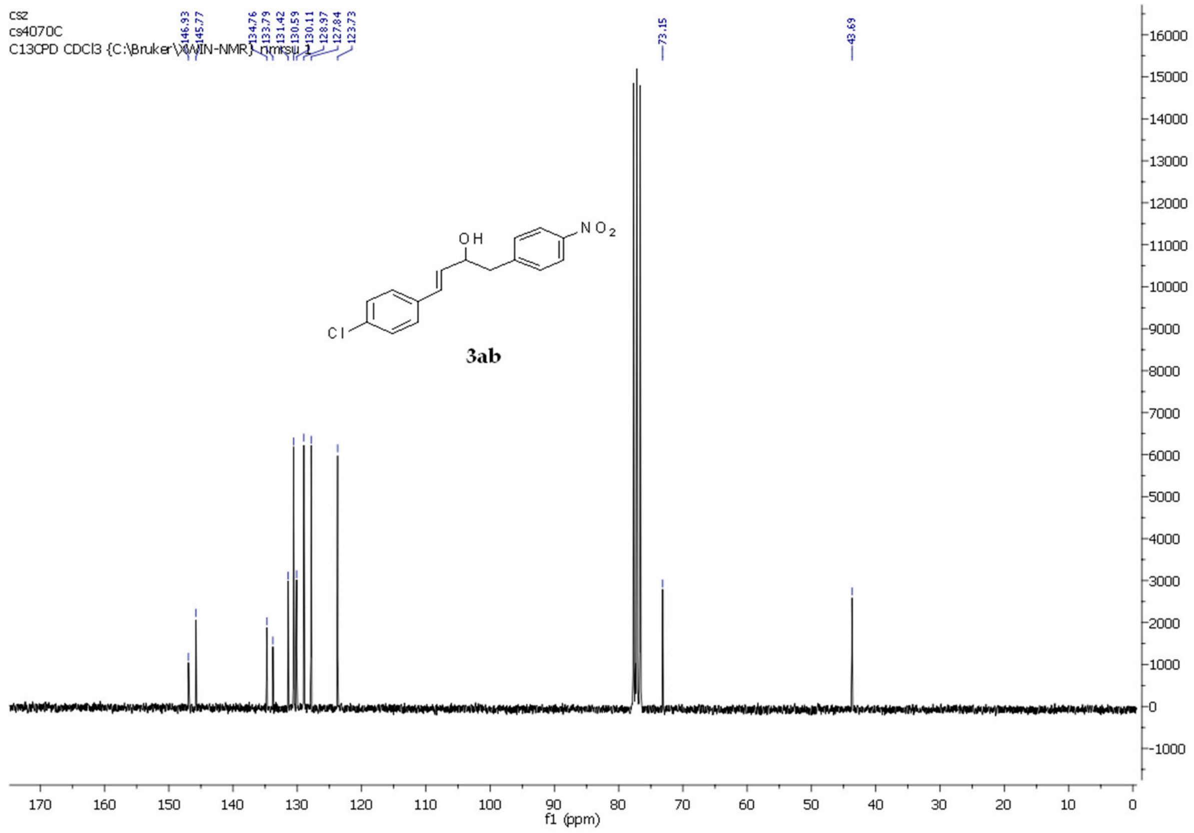

Figure S4. ^{13}C-NMR spectra of **3ab**.

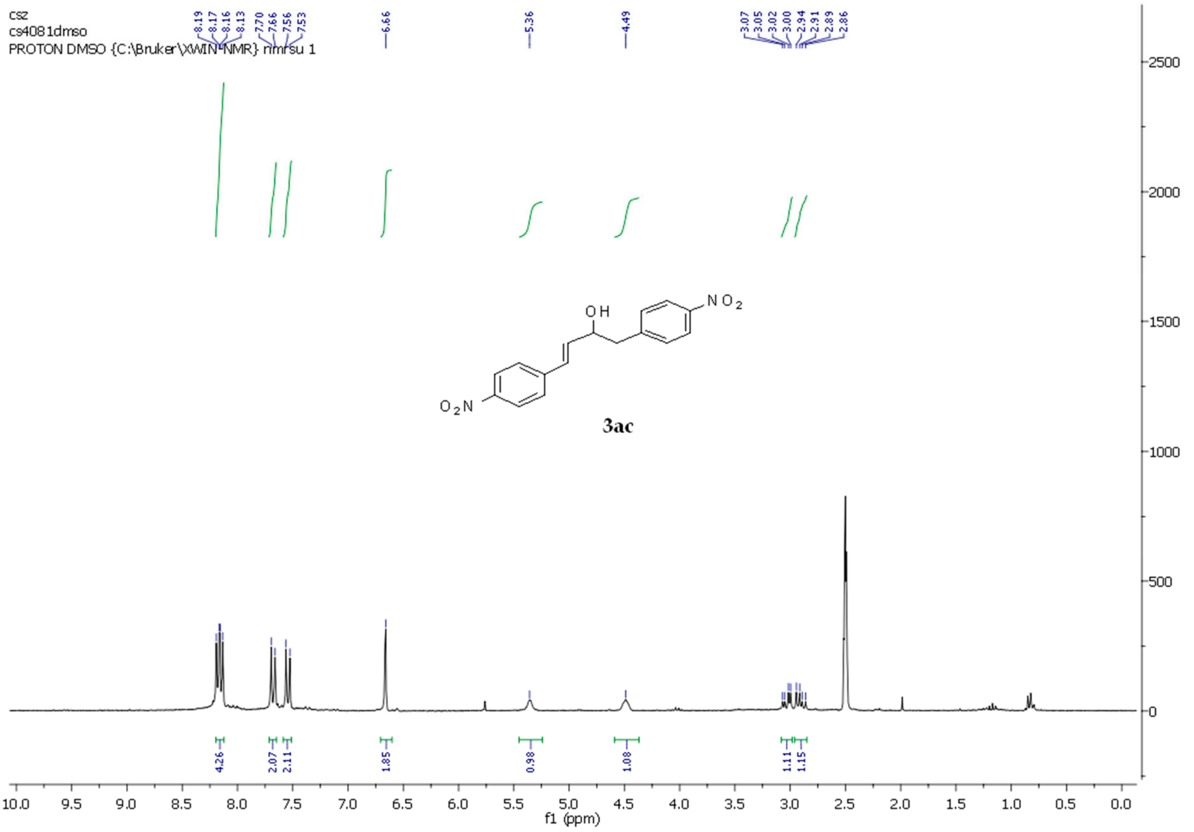

Figure S5. ^{1}H-NMR spectra of **3ac**.

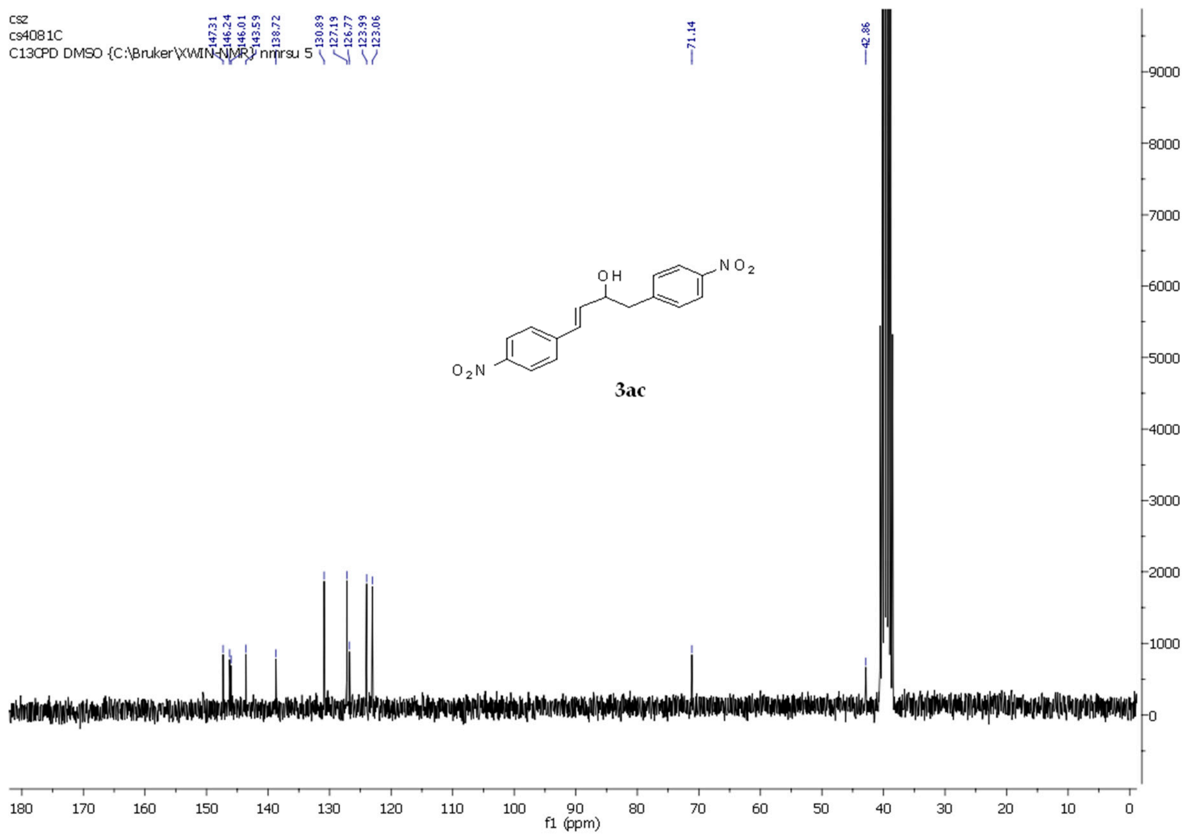

Figure S6. ^{13}C-NMR spectra of **3ac**.

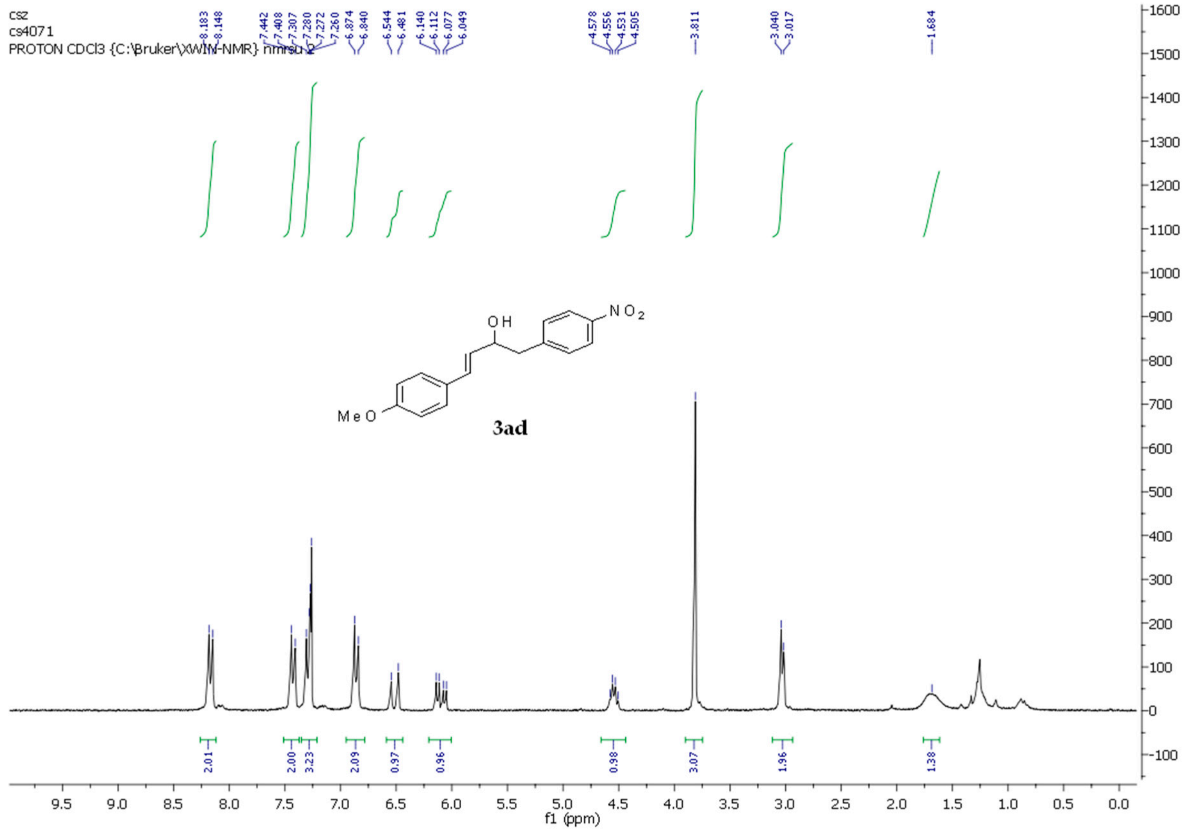

Figure S7. ^{1}H-NMR spectra of **3ad**.

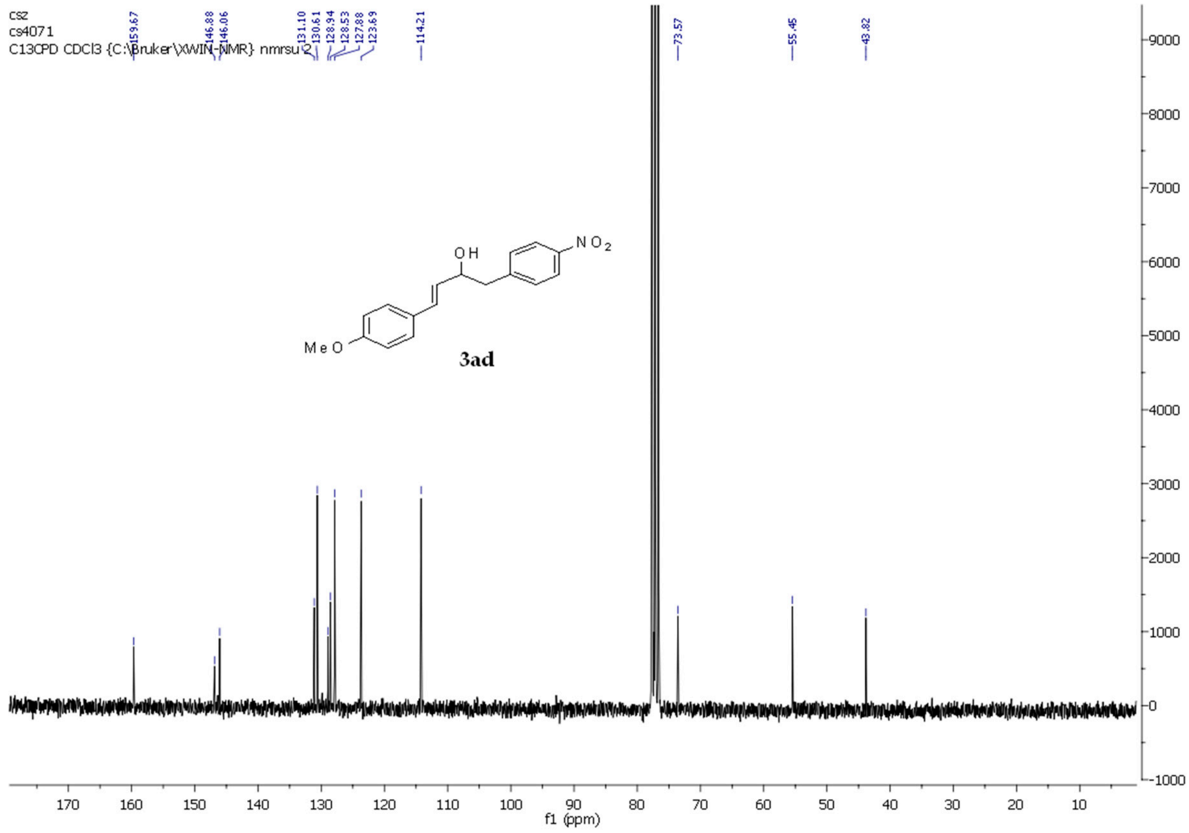

Figure S8. ^{13}C-NMR spectra of **3ad**.

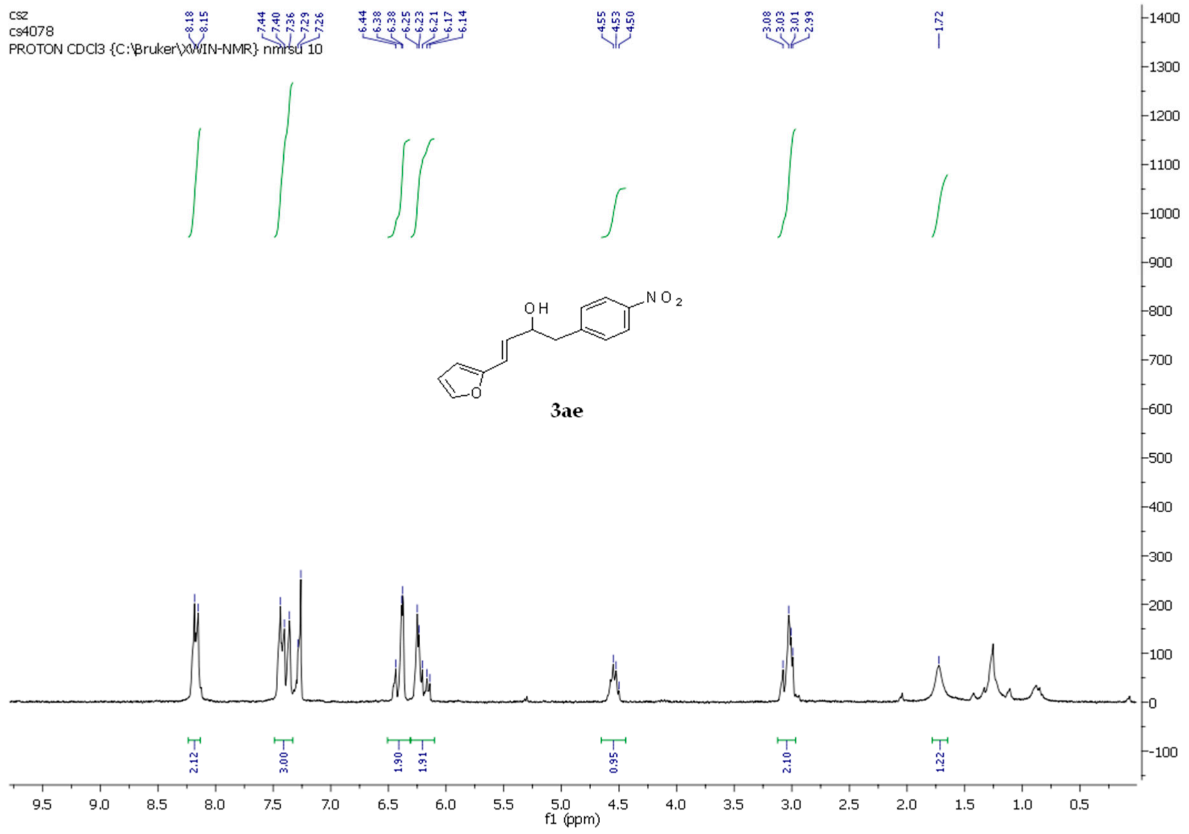

Figure S9. ^{1}H-NMR spectra of **3ae**.

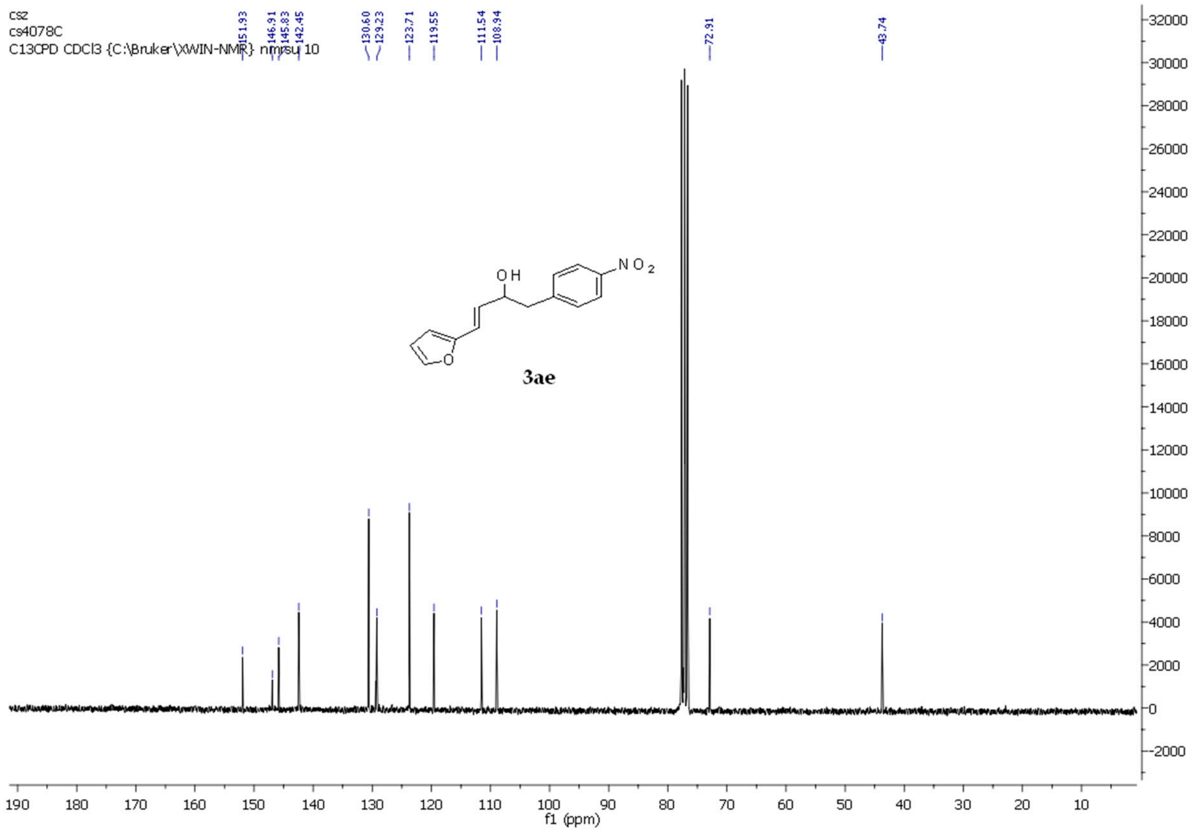

Figure S10. ^{13}C-NMR spectra of **3ae**.

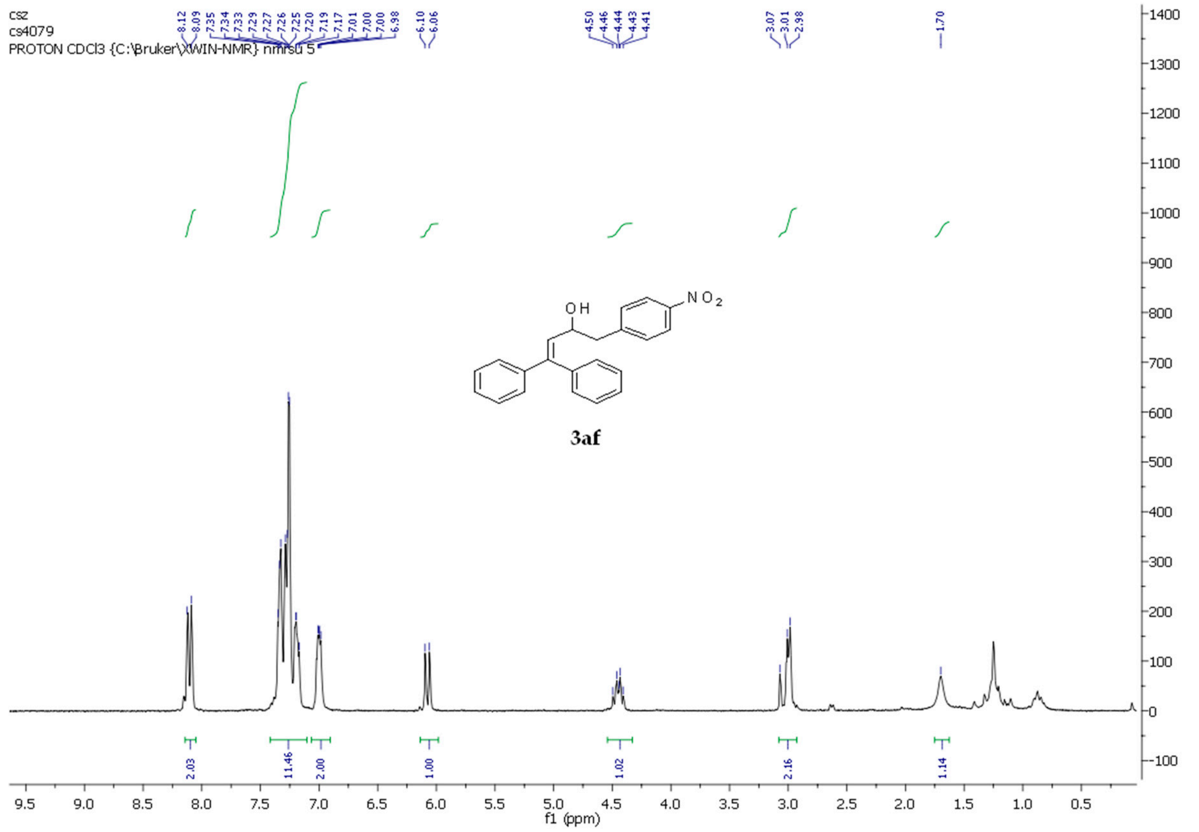

Figure S11. ^{1}H-NMR spectra of **3af**.

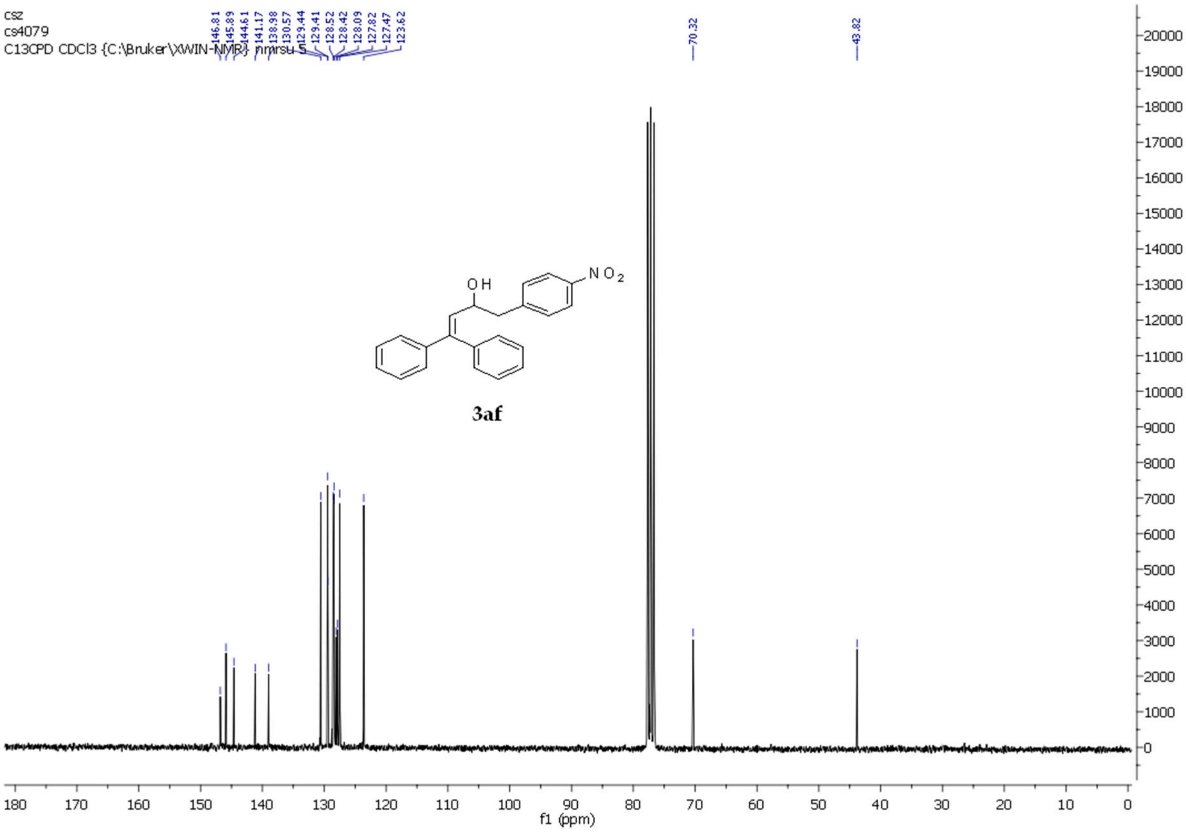

Figure S12. ^{13}C-NMR spectra of **3af**.

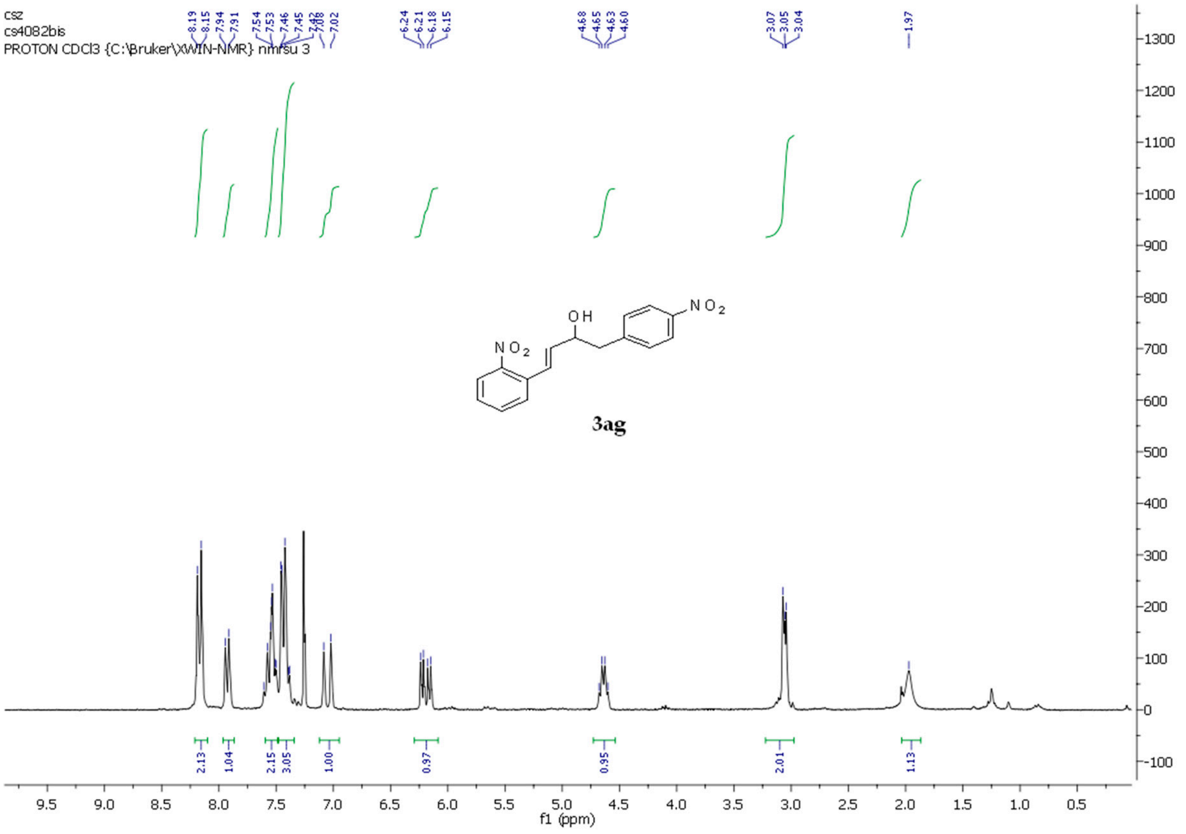

Figure S13. ^{1}H-NMR spectra of **3ag**.

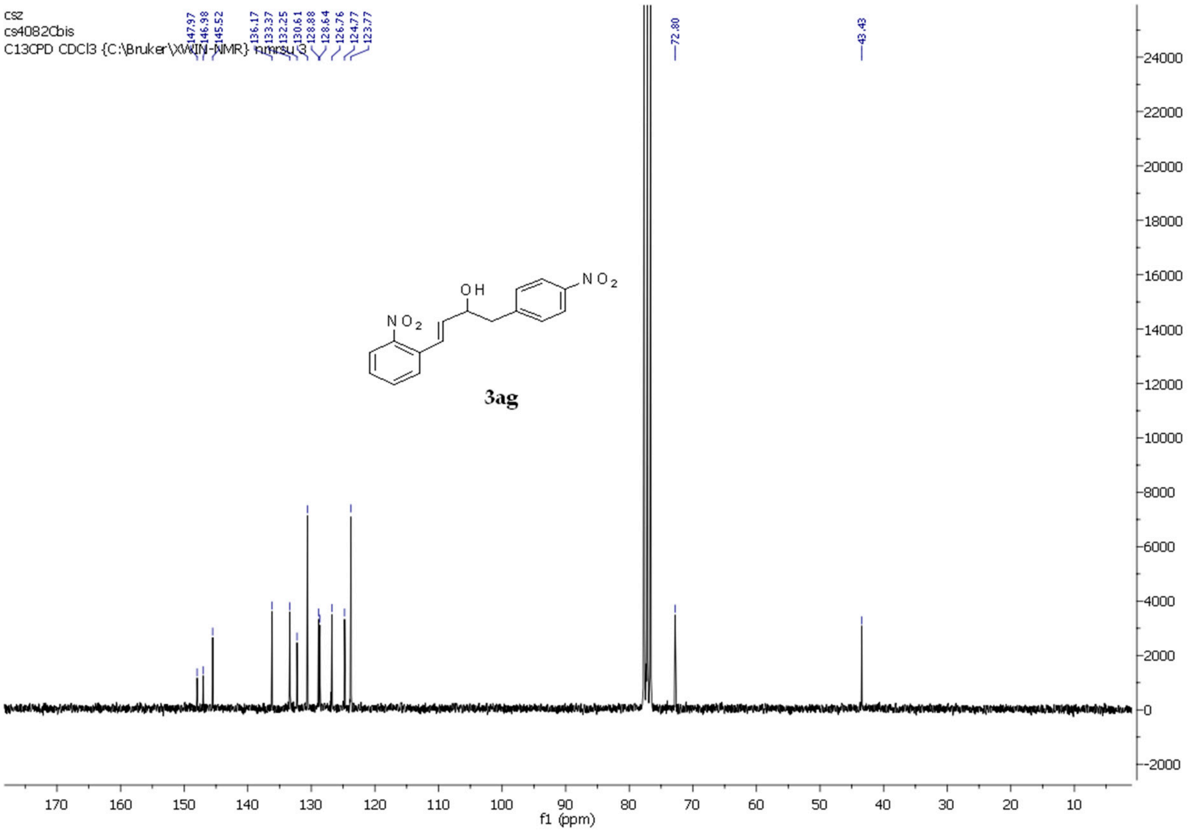

Figure S14. ^{13}C-NMR spectra of **3ag**.

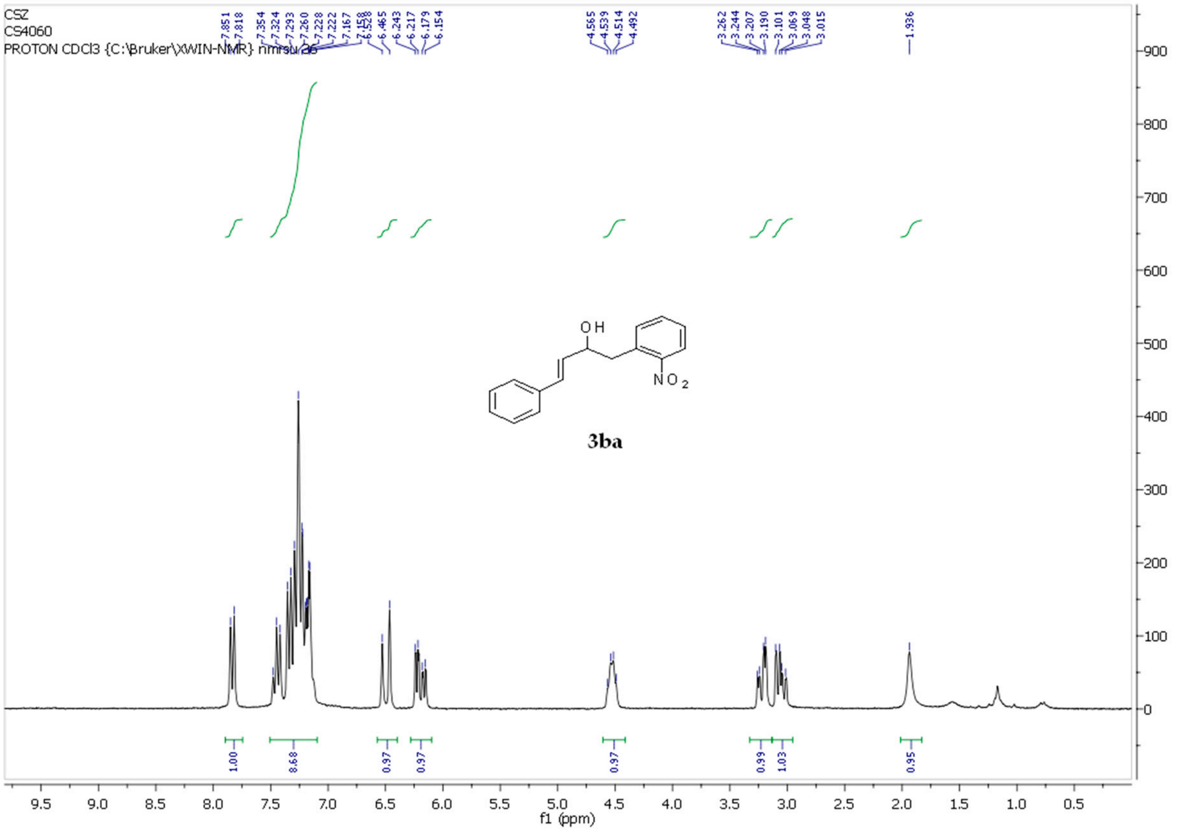

Figure S15. ^{1}H-NMR spectra of **3ba**.

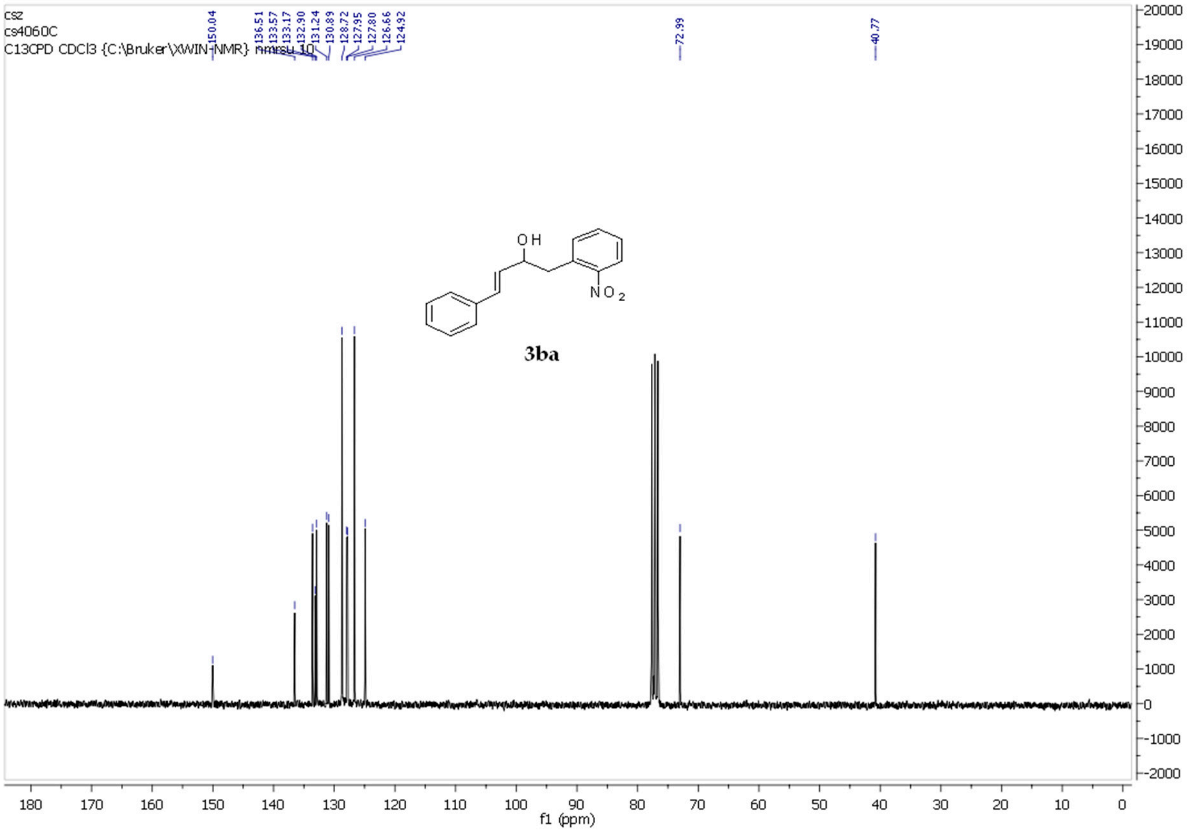

Figure S16. ^{13}C-NMR spectra of **3ba**.

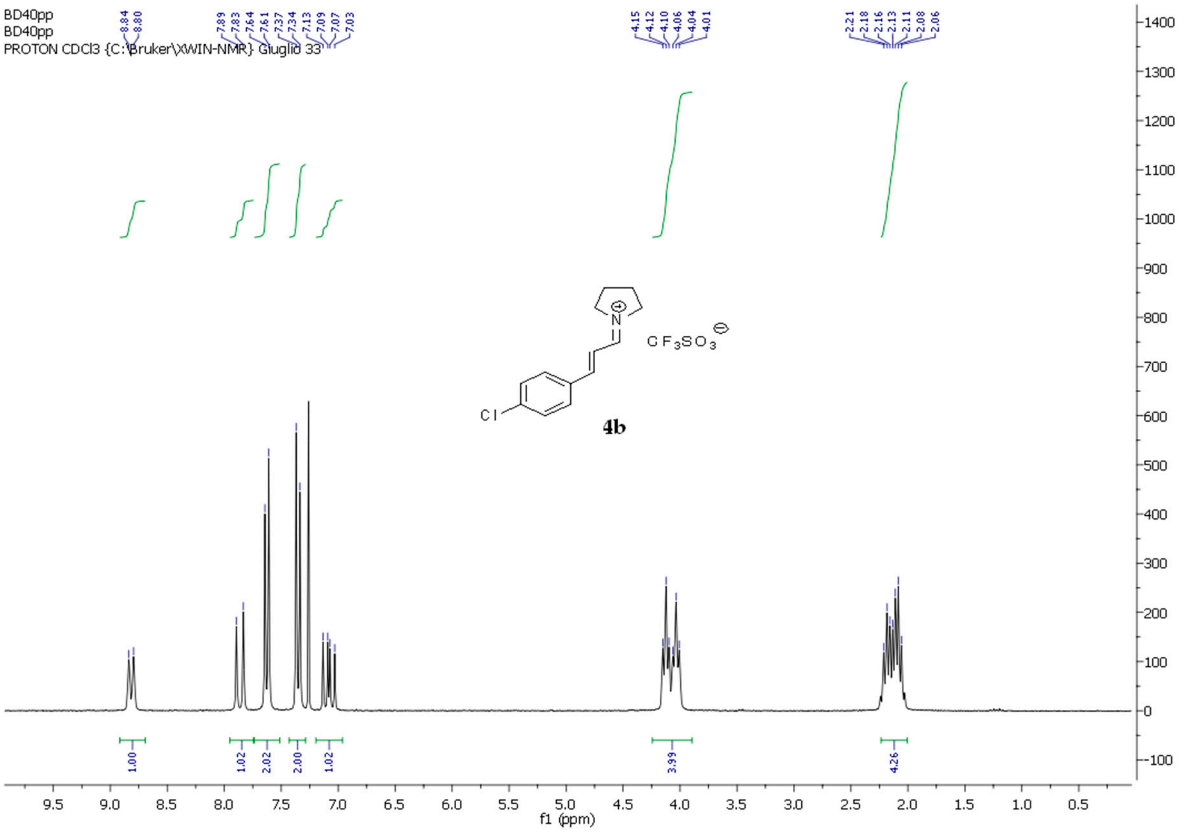

Figure S17. ^{1}H-NMR spectra of **4b**.

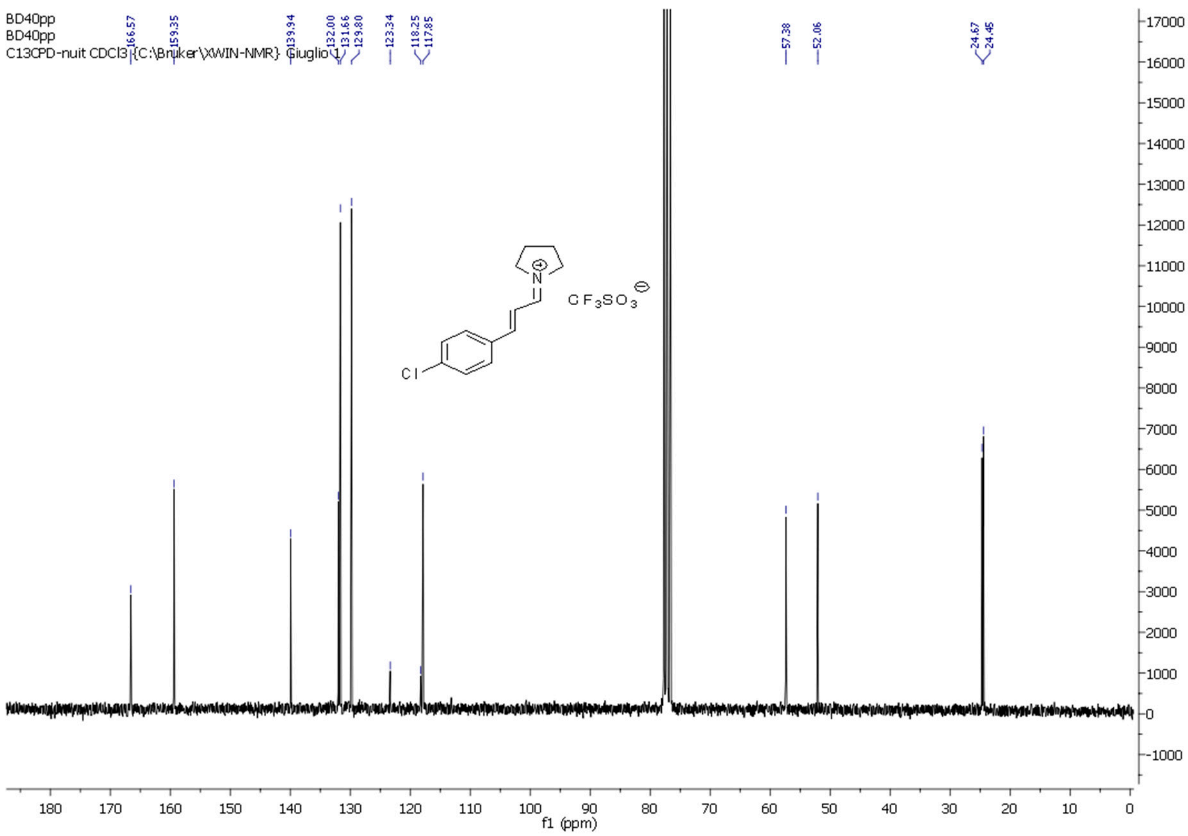

Figure S18. ^{13}C-NMR spectra of **4b**.

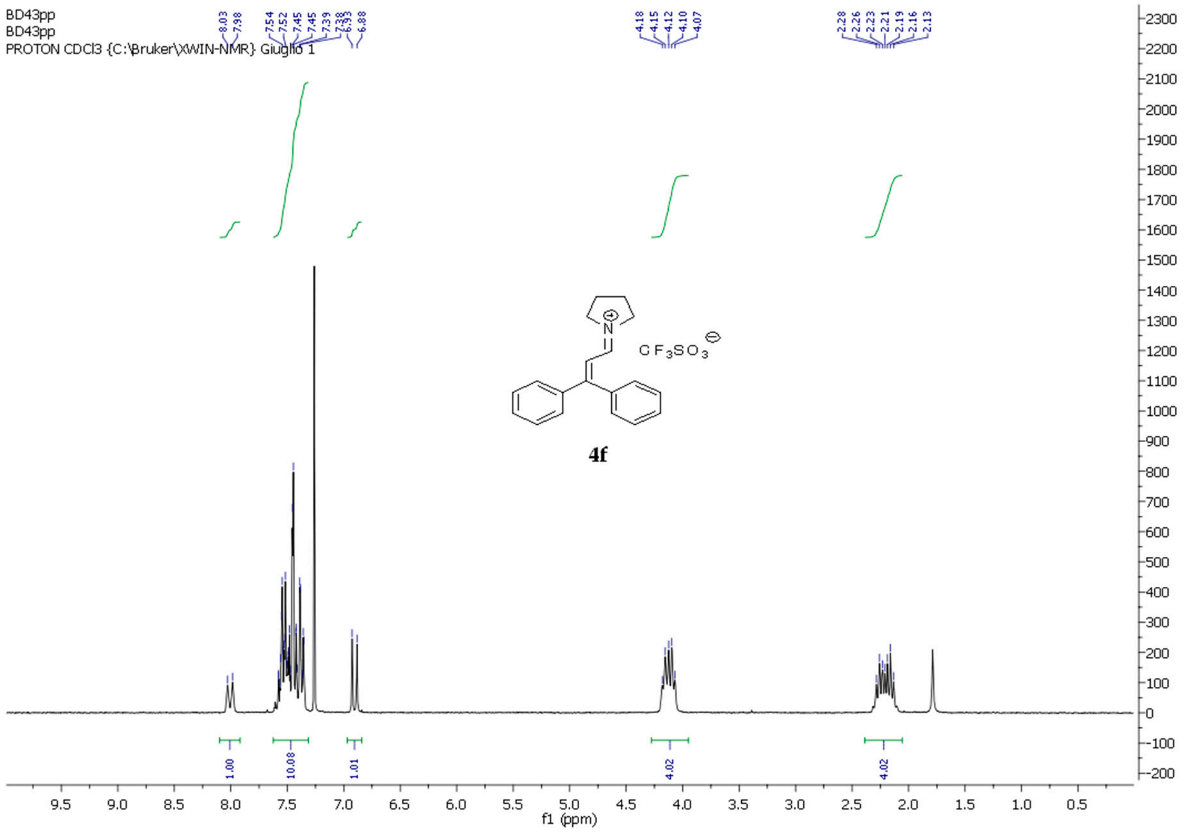

Figure S19. ^{1}H-NMR spectra of **4f**.

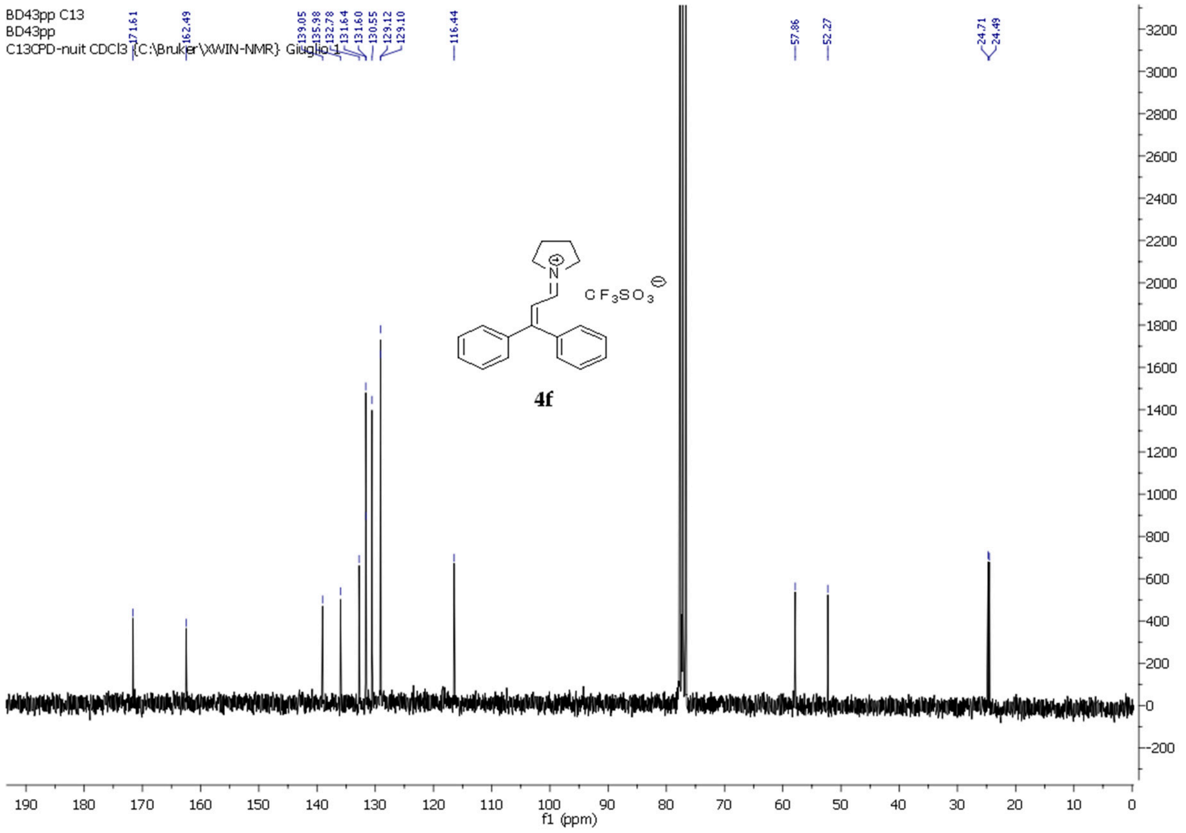

Figure S20. ^{13}C-NMR spectra of **4f**.

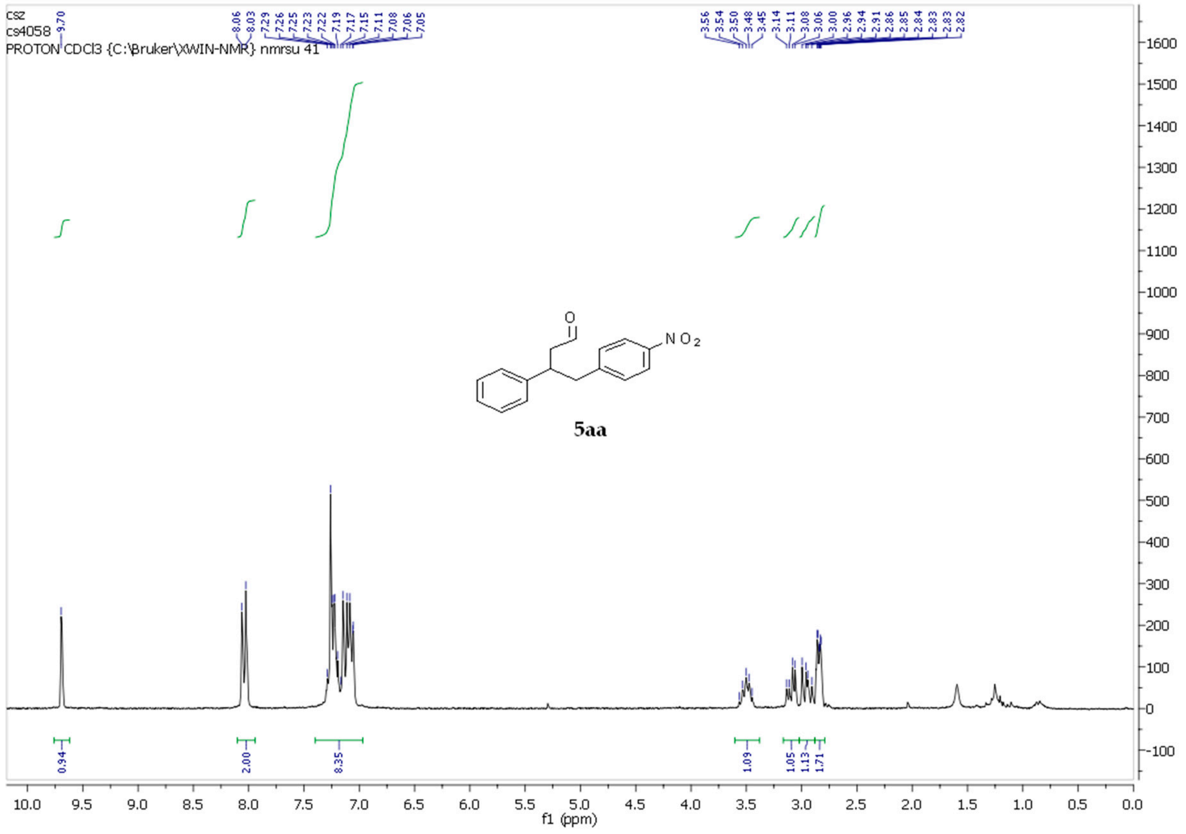

Figure S21. ^{1}H-NMR spectra of **5aa**.

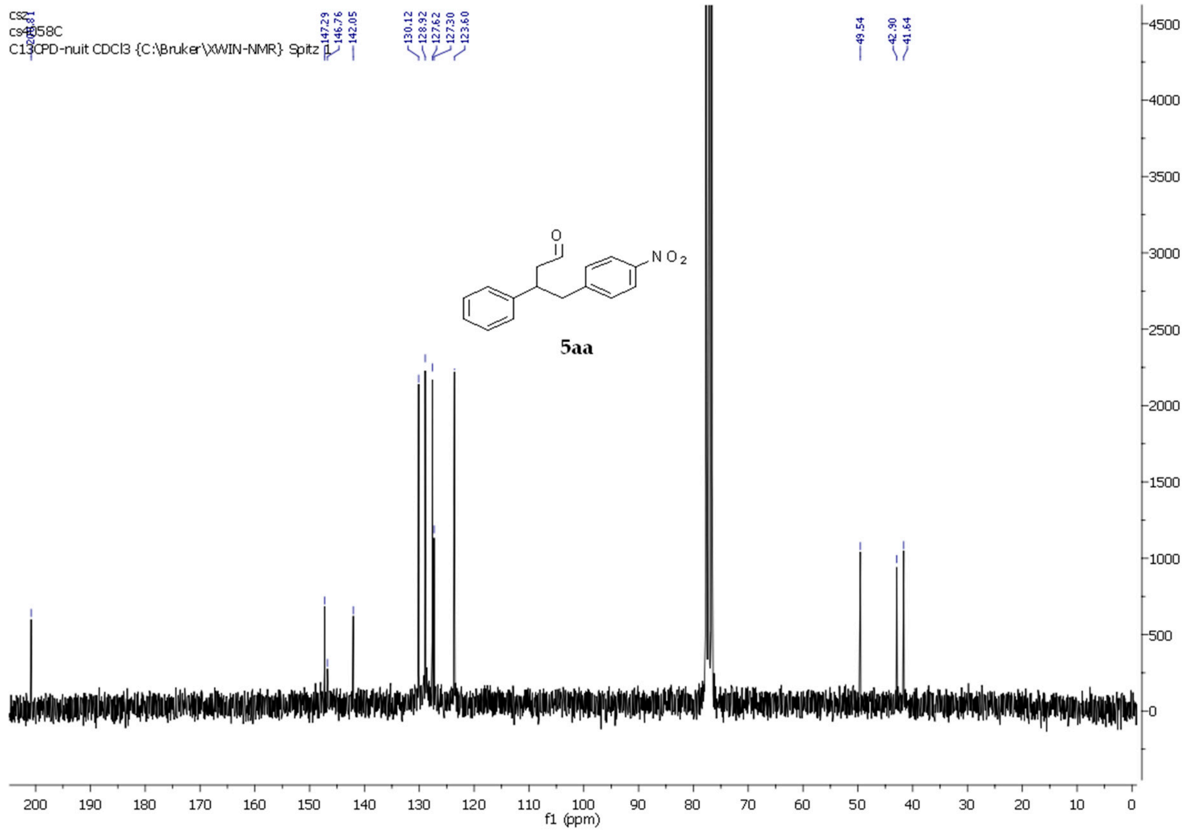

Figure S22. ^{13}C-NMR spectra of **5aa**.

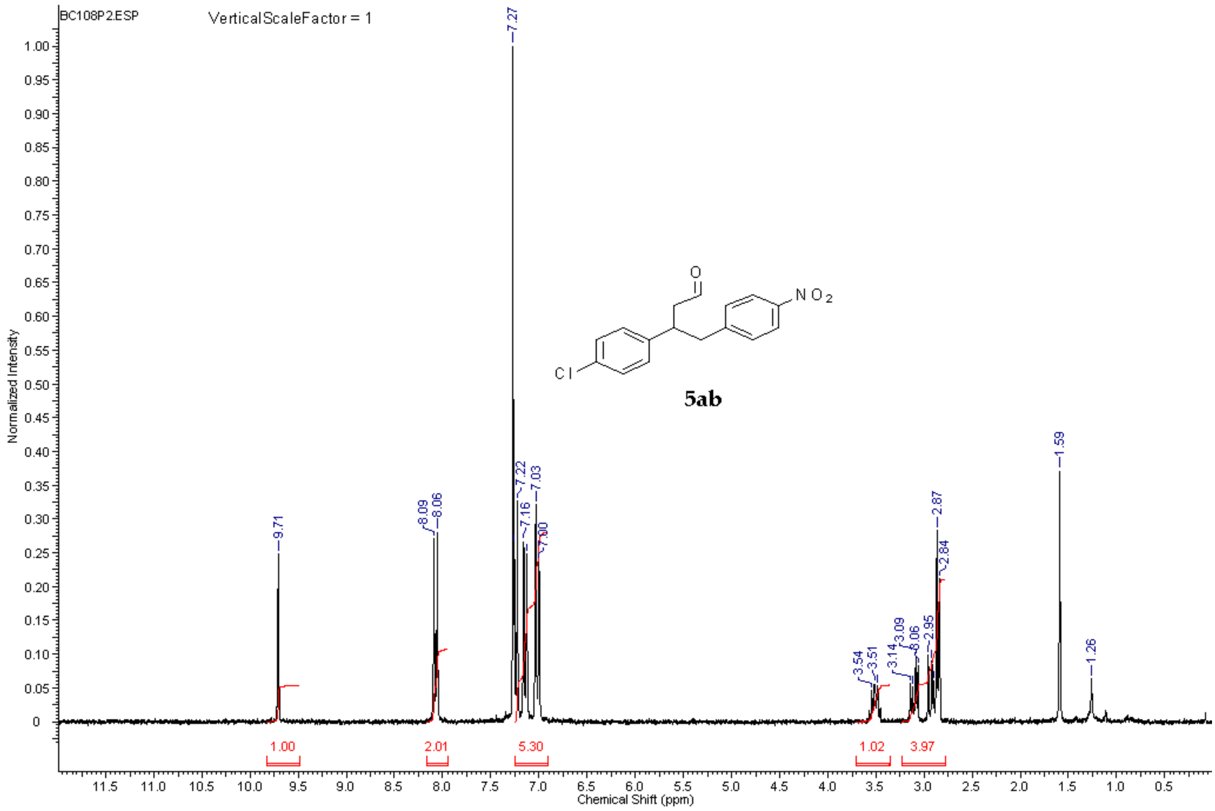

Figure S23. ^{1}H-NMR spectra of **5ab**.

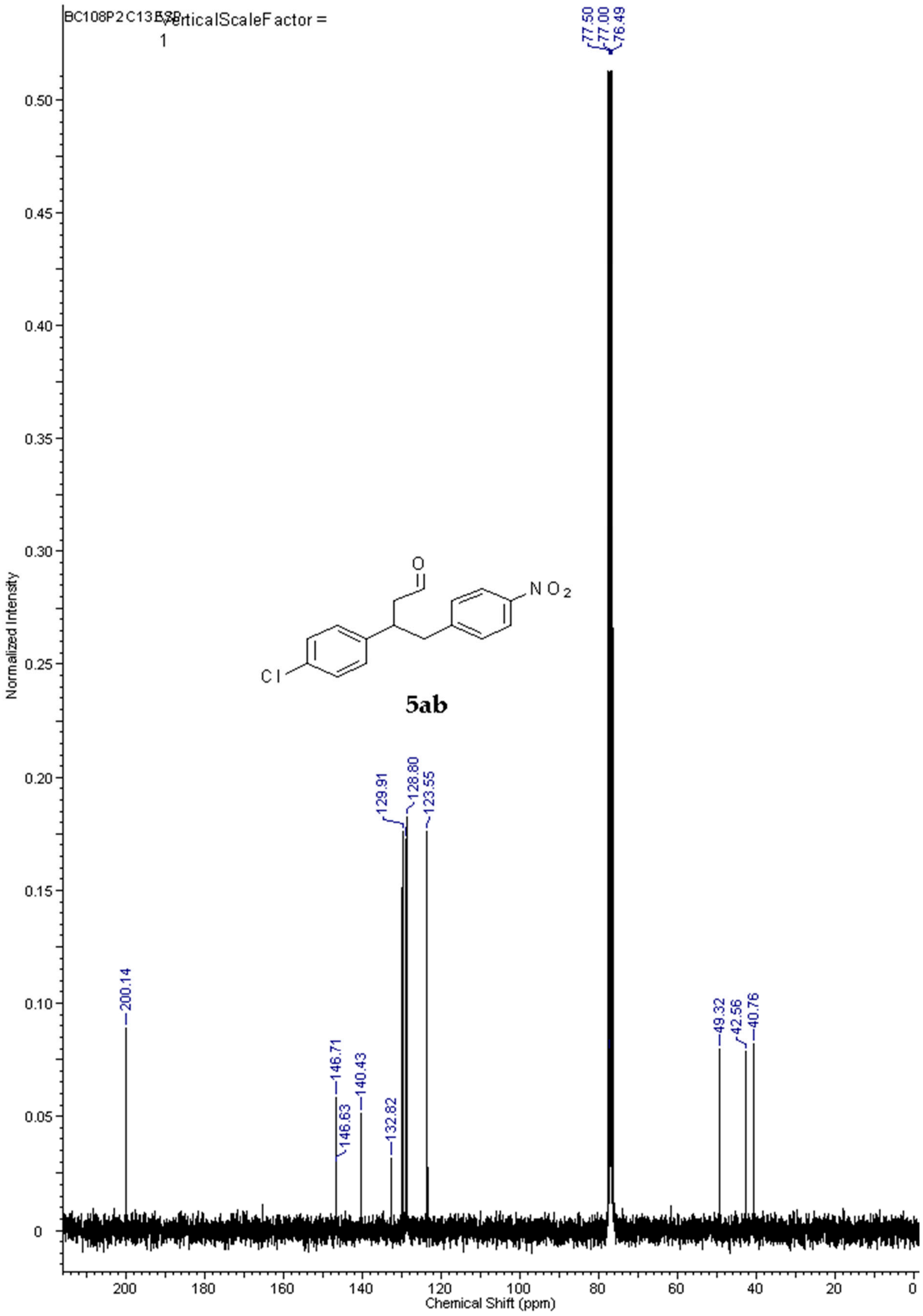

Figure S24. ^{13}C-NMR spectra of **5ab**.

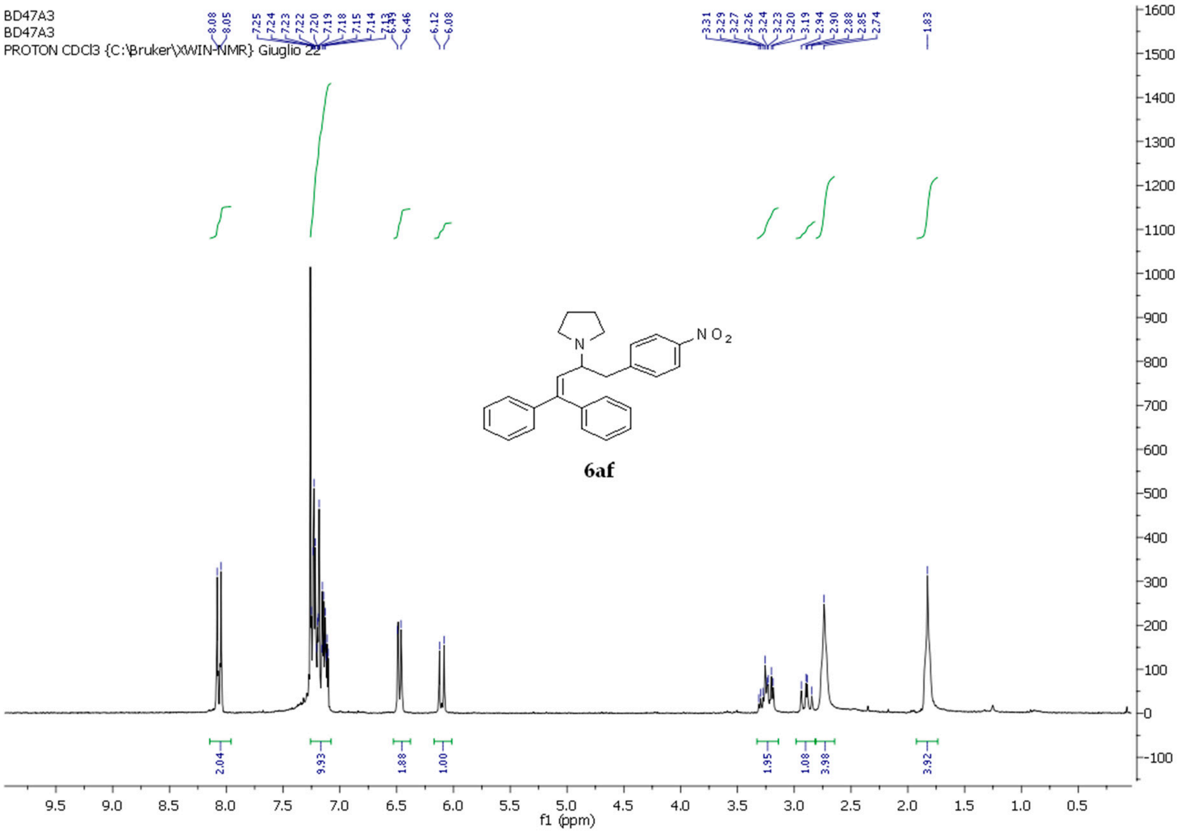

Figure S25. ^{1}H-NMR spectra of **6af**.

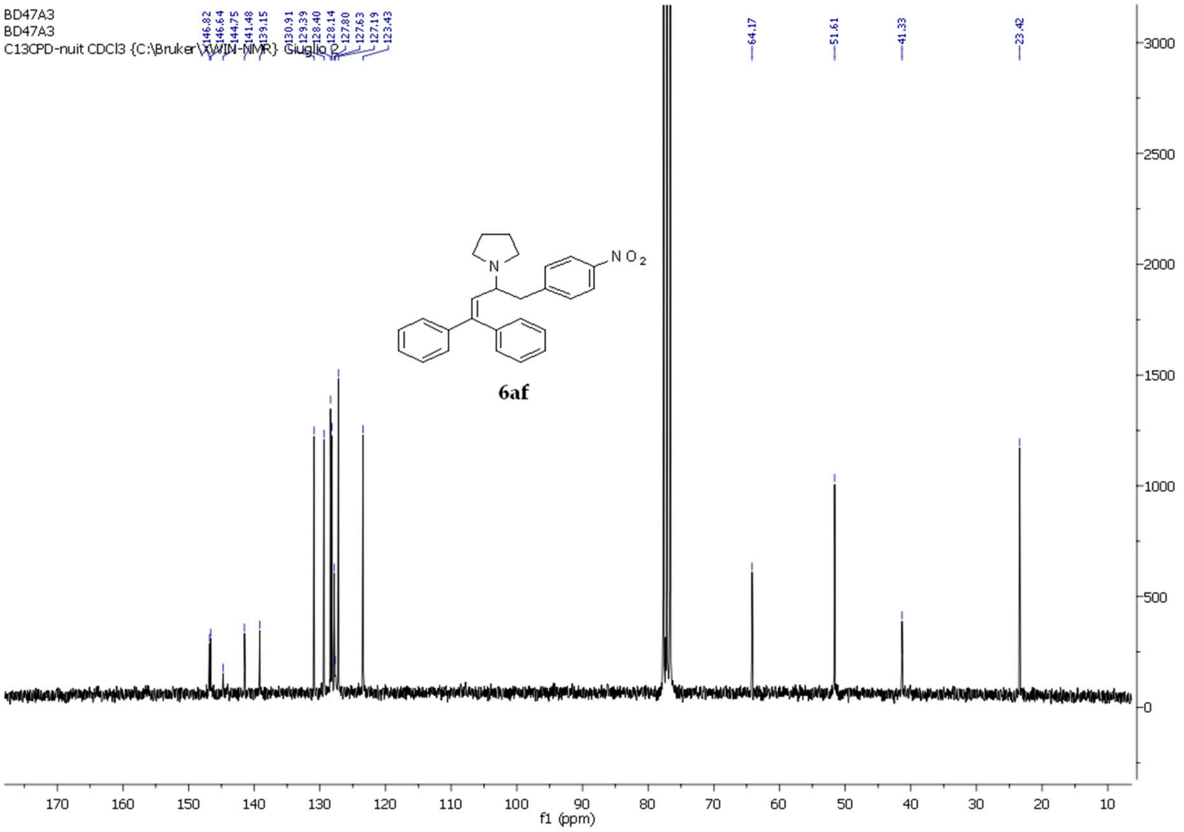

Figure S26. ^{13}C-NMR spectra of **6af**.

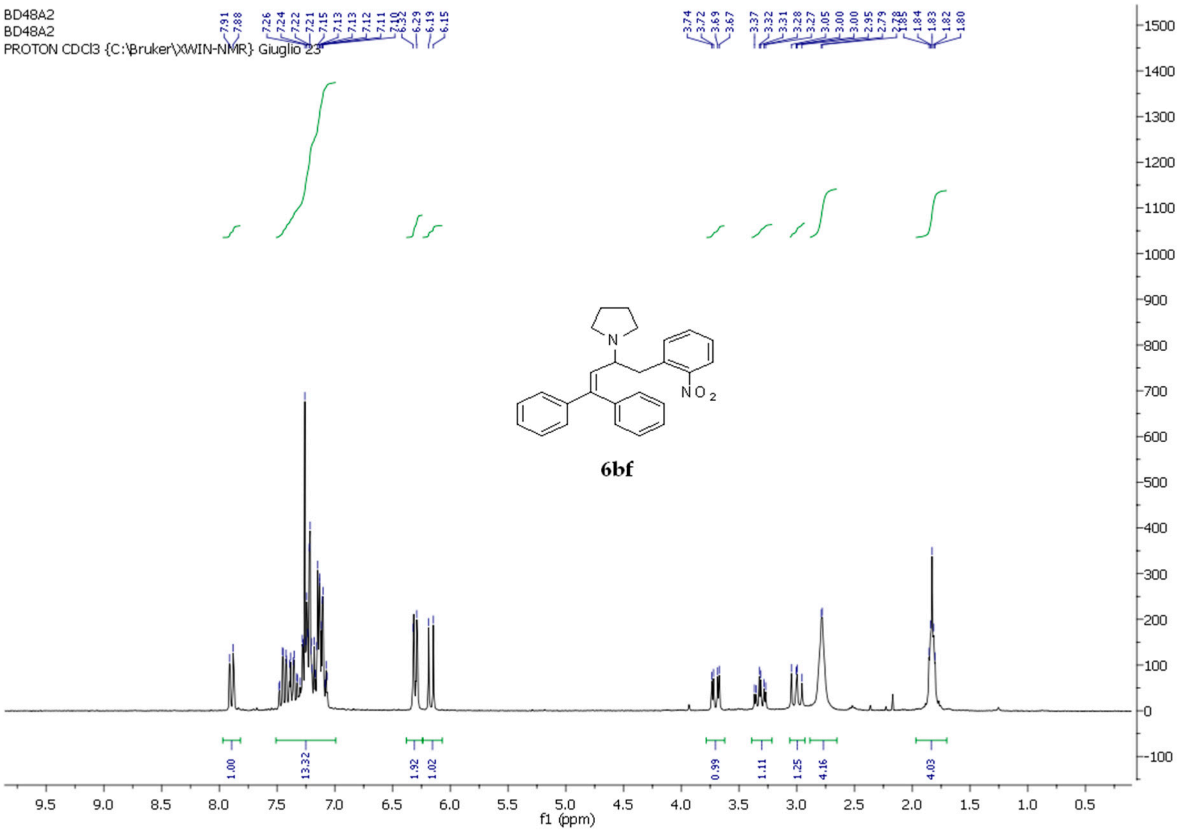

Figure S27. ^{1}H-NMR spectra of **6bf**.

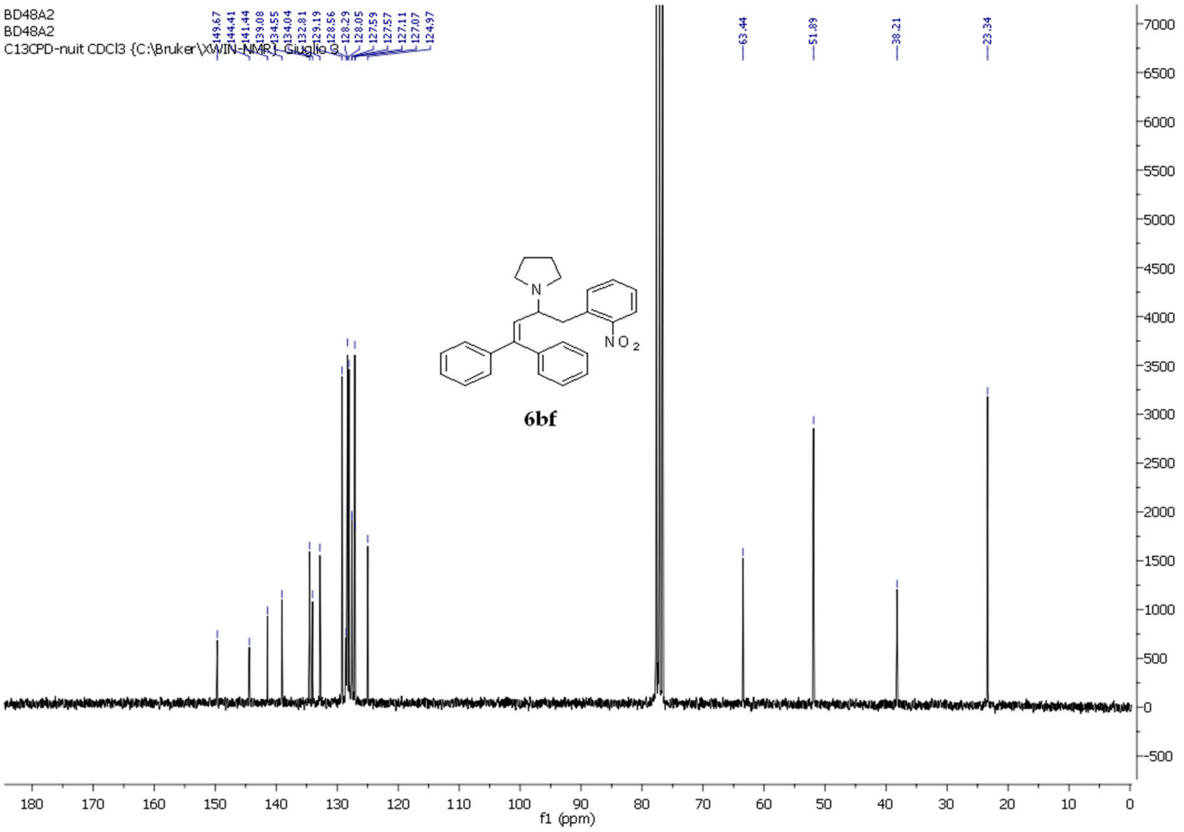

Figure S28. ^{13}C-NMR spectra of **6bf**.
